# Biogeochemical Controls on Latitudinal (42°N to 70°S) and Depth Distribution of Organophosphate Esters in the Atlantic and Southern Oceans

**DOI:** 10.1021/acs.est.4c12555

**Published:** 2025-03-11

**Authors:** Núria Trilla-Prieto, Naiara Berrojalbiz, Jon Iriarte, Antonio Fuentes-Lema, Cristina Sobrino, Maria Vila-Costa, Begoña Jiménez, Jordi Dachs

**Affiliations:** † Department of Environmental Chemistry, IDAEA-CSIC, Barcelona, Catalunya 08034, Spain; ‡ Department of Evolutionary Biology, Ecology and Environmental Sciences, Faculty of Biology, Universitat de Barcelona, Barcelona, Catalunya 08034, Spain; § Centro de Investigación Mariña (CIM), Universidade de Vigo, Vigo 36310, Spain; ∥ Grupo de Oceanografía Biolóxica, Centro de Investigación Mariña (CIM), Universidade de Vigo, Vigo 36310, Spain; ⊥ Department of Instrumental Analysis and Environmental Chemistry, IQOG-CSIC, Madrid 28006, Spain

**Keywords:** organophosphate esters, OPEs, Atlantic Ocean, Southern Ocean, plasticizers

## Abstract

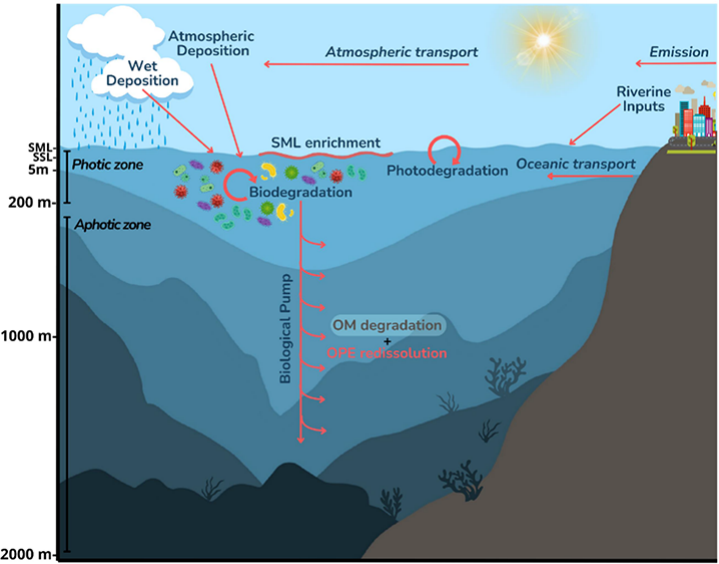

Large-scale oceanic assessments are key for determining the persistence and long-range transport potential of organic pollutants, but there is a dearth of these for organophosphate esters (OPEs), widely used as flame retardants and plasticizers. This work reports the latitudinal distribution (42°N–70°S) and vertical profiles (from the surface to 2000 m depth) of OPEs in the Atlantic and Southern Oceans and explores their biogeochemical controls. The latitudinal gradient shows higher surface OPE concentrations near the equator than at higher latitudes, consistent with the prevailing oceanic and atmospheric circulation, and measured wet deposition events. At the deep chlorophyll maximum depth, there was an inverse correlation between the concentrations of the OPEs and phytoplankton biomass, with the lowest concentrations in the Southern Ocean, consistent with the role of the biological pump depleting the levels of the OPEs from the photic zone. OPE latitudinal trends in the deep ocean (2000 m depth) resembled those at the surface with maximum intertropical concentrations. Analysis derived from OPE concentrations at the bottom of the photic zone and in the minimum oxygen layer suggested a complex dynamic biogeochemical cycling driven by transport, degradation, and redissolution of OPEs with depth. OPEs are persistent enough to reach all oceanic compartments, but a quantitative resolution of the sources, sinks, seasonality, and biogeochemical cycles will require future research.

## Abstract 2

Latitudinal trends in the Atlantic and Southern Oceans at different depths provide information on sources and biogeochemistry of the widely used organophosphate ester flame retardants and plasticizers.

## Introduction

Contemporaneous societies use hundreds of thousands of organic chemicals, and many of them reach the environment.^1^ Persistence and potential for long-range transport are two key criteria for prioritizing the regulation of persistent organic pollutants (POPs) among the myriad of synthetic chemicals. Assessments of organic contaminants at the oceanic scale, including POPs, are essential for understanding their persistence, mobility, and impacts.^2,3^ Legacy POPs,^2−7^ polycyclic aromatic hydrocarbons (PAHs),^4,8−11^ plastics^12−15^ and perfluoroalkyl substances (PFAS)^16−24^ are among the anthropogenic contaminants that have received more attention in terms of their biogeochemistry and occurrence in the ocean.

Organophosphate esters (OPEs) have been reported in many marine environments,^25−33^ but assessments at the oceanic scale are limited.^33^ OPEs are widely used as flame retardants, plasticizers, and lubricants in many consumer products and applications.^34−37^ OPEs are ubiquitous in the environment, raising concerns due to their toxicological effects,^35,36,38−40^ bioaccumulative potential,^41−44^ and persistence in ecosystems.^30,36,45−48^ Previous assessments in the marine environment report variable concentrations of OPEs,^25−28,30−32,49^ suggesting heterogeneous sources and biogeochemical pathways. For example, ∑_14_OPE concentrations ranged from 40.0 to 60.8 ng L^–1^ in the Atlantic Ocean off New York State,^50^ while in the remote Canadian Arctic,^51^ ∑_7_OPE concentrations ranged from 0.02 to 306 ng L^–1^, with drivers of such variability largely unknown. Few studies have investigated the concentration of OPE in the Southern Ocean. Li et al.^52^ reported ∑_11_OPE surface concentrations in coastal Antarctica (Fildes Peninsula, King George Island) and in the Ross Sea (1–70 ng L^–1^), which were comparable to the Arctic.^26,53^ A north–south oceanic transect of OPEs has been reported for the concentrations in the surface microlayer (SML) and subsurface layer (SSL) of the Atlantic and Southern Oceans,^54^ thus only covering the top half meter of the water column.

Even though vertical profiles of contaminant concentrations provide important information on their transport and biogeochemical cycling,^55−58^ few studies report OPEs for depths other than the top few meters of the water column. A wide range of ∑_11_OPE concentrations (0.006–0.44 ng L^–1^) were measured at five different depths (between 221 and 2513 m) in the North Atlantic’s Fram Strait, without a clear trend with depth.^29^ When evaluating biogeochemical processes, the deep chlorophyll maximum (DCM), where there is a phytoplankton biomass maxima,^59^ provides clues on the transport to deep waters by the biological pump.^19,60^ For instance, maximums of particle-phase PAHs and PCBs concentrations at the DCM in the Mediterranean Sea showed that there was a maximum of phytoplankton-bound PAHs and PCBs at the DCM.^55,56^ While the influence of the biological pump has been reported for legacy pollutants, PAHs and PFAS,^19,61−63^ it remains unexplored for OPEs. The primary known sinks of OPEs are photodegradation and hydrolysis, the former may occur predominantly close to the surface,^64−66^ as well as microbial degradation.^67^ Other regions in the mesopelagic ocean, such as the layer with a minimum of oxygen, remain unexplored in terms of the biogeochemistry of contaminants. Furthermore, it is unclear how the occurrence at the surface relates to the other depths of the water column, which is an important issue as most oceanic measurements are made at 4–5 m depth.^25−28,30,31,49^

OPEs exhibit a broad spectrum of physicochemical properties, such as volatility, aqueous solubility, and octanol–water partition coefficients (*K*_OW_), attributed to the varying substituents on their side chains.^68^ Such variability in properties and potential for being degraded may introduce variations in their biogeochemistry and eventually their oceanic sinks, even though *K*_ow_ is often a poor descriptor for polar chemicals. Measurements in large-scale transects and vertical profiles are useful to identify regions under a larger influence of sources (rivers, atmospheric inputs including rain), potential for long-range transport, and cycling processes. The wet deposition has barely been quantified for OPEs in the open ocean, as previous measurements focused on continents and coastal zones, even though rain-air washout ratios are high.^69^

Here, we provide a large data set of concentrations for 24 targeted OPEs in the dissolved phase at 5 m depth and the DCM covering a latitudinal transect from 42°N to 53°S in the Atlantic Ocean, and from 60°S to 70.2°S in the Southern Ocean. In addition, we provide 11 vertical profiles of concentrations from the surface to 2000 m depth. The objectives of this work are (a) to describe the latitudinal and depth distribution of OPEs in the Atlantic and Southern Ocean, (b) to assess the biogeochemical controls on the latitudinal and depth occurrence of OPEs, and (c) to provide measurements of wet deposition of OPEs over the ocean.

## Materials and Methods

### Sampling

**Figure 1:**
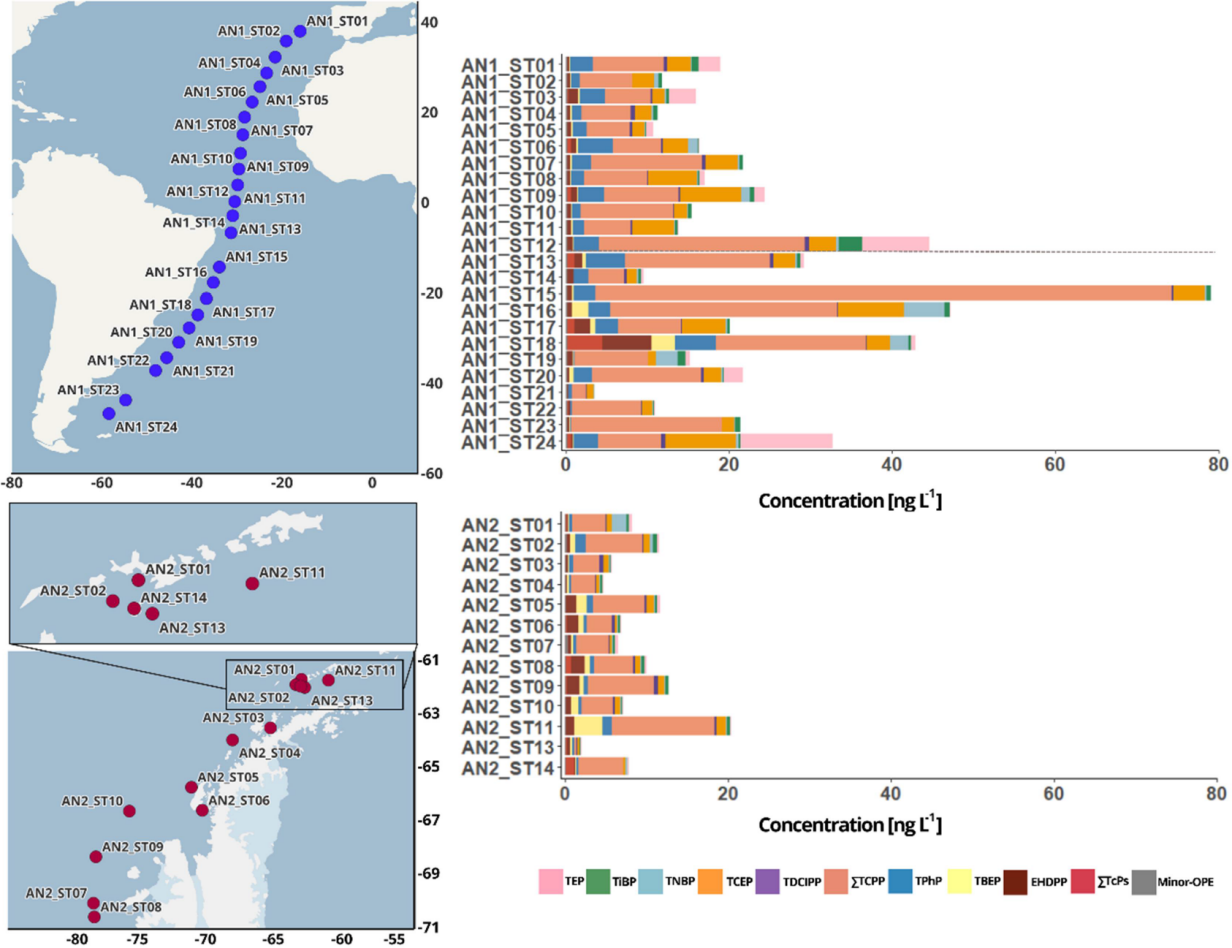
Latitudinal variability of OPEs at 5 m depth. Top left panel shows location of stations in the Atlantic Ocean; top right panel shows 5 m depth concentrations of individual and ∑_24_OPE (ng L^–1^) in the Atlantic Ocean; bottom left panel shows location of stations in the Southern Ocean; and bottom right panel shows 5 m depth concentrations of individual and ∑_24_OPE (ng L^–1^) in the Southern Ocean.

Seawater from 5 m depth (below the influence of the ship) and at additional depths down to 2000 m, as well as wet deposition samples were collected during two oceanographic expeditions. During the ANTOM-I cruise (December 2020–January 2021), from Vigo (Spain) to Punta Arenas (Chile), a total of 24 stations were sampled along a latitudinal transect in the Atlantic Ocean onboard the R/V Sarmiento de Gamboa. The second campaign, ANTOM-II onboard the R/V Hespérides (January to February 2022), covered the Southern Ocean, west of the Antarctic Peninsula, sampling at 13 stations in the Bransfield Strait and the Bellinghausen Sea (Table S1). Seawater samples were collected using a Niskin-rosette system equipped with a Sea-bird SBE 9 CTD, which measured salinity, temperature, fluorescence, depth, and oxygen concentrations, enabling the characterization of the water column. The 5 m depth water samples were collected at all the stations (*N* = 37). In addition, seawater from the DCM, determined using the fluorescence sensor from the CTD, was sampled at 16 stations in the Atlantic Ocean, and 10 stations in the Southern Ocean. Vertical profiles (5 depths) were obtained at 7 stations in the Atlantic Ocean and 4 stations in the Southern Ocean. These depth profiles included samples, in addition to surface and DCM, from the layer receiving 1% of photosynthetic active radiation (1% PAR), the layer with a minimum oxygen concentration (MOx), and at a depth of 2000 m. At 2 stations from the Southern Ocean (AN2_ST08 and ST11, see Figure 1), intense water mixing disrupted the oxygen gradient, and only a weak minimum was observed.

Two liters of seawater were collected from Niskin bottles into pre-rinsed 2 L polytetrafluoroethylene (PTFE) bottles, and seawater was preprocessed in the vessel’s laboratory. First, seawater was filtered through precombusted glass fiber filters (47 mm, GF/F Whatmann). The dissolved phase was then collected using Oasis HLB solid-phase extraction (SPE) cartridges (6 cm^3^, 200 mg; Waters). The cartridges were preconditioned with 6 mL of 2-propanol and 12 mL of HPLC-grade water and then spiked with 50 ng of a mix of labeled recovery standards (Table S2). After sample loading, cartridges were washed with 6 mL of HLPC-grade water containing 5% methanol, dried under vacuum for 10 min, and stored at −20 °C in sealed bags until processed ashore in a clean laboratory.

During the only two rain episodes that occurred during the campaigns, wet deposition (WD) samples were collected using a precombusted 2.5 L glass bottle and a MeOH and acetone precleaned stainless-steel funnel (diameter of 0.2 m). The WD samples were treated equally to the rest of the seawater samples, with the exception of the GF/F filtering step, which was not performed. Two samples were collected from the Atlantic Ocean (station 10 at latitude 7°N, and 11 at latitude 4°S). The first was collected during the night (8 h) from December 26 to 27th 2020, and the second was collected the following night (8 h), from December 27 to the 28th. For both rain events, 1 L of rain was analyzed, but during the rain events, 1 and 1.2 L of rainwater were collected. There were no WD events during the Southern Ocean campaign.

### OPE Analysis

After transport of the samples to the clean laboratory, HLB cartridges were unfrozen for 3 h at room temperature, centrifuged at 3500 rpm for 4 min, and eluted with 12 mL of methyl *tert*-butyl ether:methanol (3:1, v/v). Residual water was removed from the extracts by adding 3 g of prebaked sodium sulfate. The final eluents were concentrated under N_2_ avoiding dryness and reconstituted in 300 μL of toluene.

OPE identification and quantification were conducted using gas chromatography coupled to a triple quadrupole mass spectrometer (GC-MS/MS-QqQ). Chromatographic separation was carried out using an HP-5MS column (30 m, 0.25 mm internal diameter, 0.25 μm film thickness) on an Agilent 7890A gas chromatograph. Methane was used as the ionization gas, and helium was used as the carrier gas at a constant flow rate of 20 mL min^–1^. Two microliters of sample were injected in splitless mode at an injection port temperature of 280 °C. The column temperatures were: 90 °C for 1 min, increased at 15 °C min^–1^ to 200 °C, held for 6 min, then at 5 °C min^–1^ to 250 °C, held for 6 min, and then at 10 °C min^–1^ to 315 °C, held for 10 min. Detection was carried out with an Agilent 7000B triple quadrupole mass spectrometer using the electronic impact ionization (EI) mode in positive mode. Acquisition was performed by multiple reaction monitoring (MRM). Fifty nanograms of internal standards (Table S2) were added before the analysis. Concentrations were recovery corrected and only concentrations above limits of quantification (LOQ) were considered.

A total of 24 OPEs were targeted in this study (acronyms in Table S3). These include five chlorinated OPEs (TCEP, TDCIPP, and three TCPP isomers), one brominated OPE (TDBPP), eight aryl OPEs (TPhP, EHDPP, ToCP, TpCP, TmCP, TDMPP, TPPP, and TTBPP), and six alkylated OPEs (TEP, TPrP, TiBP, TNBP, TBEP, and TEHP). In addition, four organophosphorus chemicals were targeted: TPPO, DOPP, Chlorpyrifos, and TBPO. For this analysis, the three TCPP isomers were grouped together as ∑TCPP, and the three cresyl isomers (TmCP, ToCP, and TpCP) were grouped as ∑TcPs. Furthermore, analytes contributing less than 0.5% on average to the total OPE pool were grouped under the name “Minor-OPEs”, which included TEHP, TPrP, TPPP, DOPP, TBPO, TPPO, Chlorpyrifos, TDMPP, TDBPP, and TTBPP (see Table S4). For simplification, the term “OPEs” in the discussion encompasses all targeted organophosphorus compounds, and (i) Cl-OPEs include TCEP, ∑TCPP, and TDCIPP; (ii) aryl-OPEs include TPhP, EHDPP, and ∑TcPs; and (iii) alkyl-OPEs includes TEP, TiBP, TNBP, and TBEP.

### Quality Assurance and Quality Control

All containers, tubes, and connections used during sampling and chemical analysis were made of stainless steel, glass, or PTFE, and they were rinsed with methanol and acetone to avoid contamination. To minimize contamination, all filters and glass materials were combusted at 450 °C for 4 h before their use.

A minimum of a field blank and a procedural blank were analyzed for each batch of 10 samples to monitor potential contamination from the laboratories and during transport. Field blanks consisted of HLB cartridges that followed the same preconditioning, transport, and analysis as the samples, but no seawater was loaded into the cartridges even though they were connected to the SPE system for the same time as the sample cartridges to replicate handling conditions. Procedural blanks followed the same extraction and analysis procedures as those conducted in the clean laboratory. The limits of quantification (LOQs) were defined as the mean concentrations of field/procedural blanks (Table S5) plus three times the standard deviation (Table S6). The recoveries were monitored using TNBP-d27, TCEP-d12, TCPP-d18, TEHP-d51, and TPhP-d15 as surrogate standards added to the cartridge prior to the loading of the sample, with mean recoveries of 71% ± 44% and 74% ± 20% in the Atlantic and Southern Ocean, respectively (Table S7). Six and four matrix spikes were performed during the Atlantic and Southern Ocean campaigns, respectively. The recoveries of the native analytes ranged from 18.3% ± 2.8% to 144.6% ± 13.2% in the Atlantic, and from 41.4% ± 1.2% to 113.8% ± 8.7% in the Southern Ocean (Table S7).

### Biological Measurements

Bacterial abundance (BA), bacterial production (BP), and chlorophyll *a* (Chl *a*) were also measured to provide further information about the biological interaction between marine microbial plankton assemblages and OPEs (see Note S1 for methods).

### Statistical Analysis

Due to the non-normal distribution of concentrations (confirmed by Shapiro-Wilk normality test, *p* < 0.01), nonparametric statistical analysis, or log-transformation of the data was conducted using RStudio 2023.12.1+402. The Mann–Whitney *U* test and Kruskal–Wallis test were used to detect significant differences between independent groups, whereas the Wilcoxon Signed-Rank test was used for paired comparisons. Additional analyses included the utilization of polynomial and linear regression models, and Spearman, and Pearson’s correlation tests.

## Results and Discussion

### Latitudinal Distribution of OPEs at 5 m Depth

The dissolved phase concentrations of ∑_24_OPE at 5 m depth ranged from 2.12 to 78.5 ng L^–1^ in the Atlantic Ocean (AN1_ST01 to ST24) with an average of 23.4 ng L^–1^ (Tables S8 and S9 for complete statistics). In the Southern Ocean (stations AN2_ST01 to ST14), 5 m depth ∑_24_OPE concentrations were significantly lower, ranging from 1.42 to 20.1 ng L^–1^, averaging 9.14 ng L^–1^ (Tables S9 and S10). The highest ∑_24_OPE concentrations at 5 m depth were recorded along the Brazilian coast at stations AN1_ST15, AN1_ST16, AN1_ST12, and AN1_ST18 (Figure 1). This spatial pattern aligns with rivers’ influence, such as the Amazon, identified as a significant source of OPEs in the tropical Atlantic Ocean. High dissolved-phase concentrations of OPEs, reaching up to 1 μg L^–1^, have been reported within the Amazon River plume.^30^

In the Atlantic Ocean, the OPE patterns at 5 m depth were dominated by ∑TCPP, TCEP, and TPhP, the first particularly prevalent at most stations. Previous studies for seawater, air, and sediment compartments have highlighted the predominance of Cl-OPE within the total OPE pool,^26,29,70,71^ which is consistent with the large production of TCEP and TCPPs, and Cl-OPEs being less prone to degradation than alkyl and aryl-OPE by photolysis and/or hydrolysis.^65,66,72^ In the Southern Ocean, the OPE profile was dominated by ∑TCPP, EHDPP, and TBEP, suggesting different sources and potentially distinct biogeochemistry in the water column.

Several studies have documented OPE concentrations in surface seawater (measured at 4–5 m depth), highlighting a large variability across marine regions. For example, Xie et al.^49^ reported ∑_14_OPE concentrations ranging from 7.65 to 270 ng L^–1^ in the Pearl River Estuary and South China Sea. In the North Atlantic, near the Amazon River plume, Schmidt et al.^30^ observed ∑_9_OPE concentrations between 74 and 1340 ng L^–1^. Similarly, in Marseille Bay (NW Mediterranean Sea), ∑_9_OPE concentrations ranged from 9 to 1013 ng L^–1^.^31^ Bollmann et al.^25^ found ∑_16_OPE concentrations in the North Sea ranging between 5 and 50 ng L^–1^, while Na et al.^32^ measured ∑_11_OPE concentrations between 8.47 and 143.4 ng L^–1^ from the Northwestern Pacific to the Arctic Ocean. In the North Atlantic and Arctic, Li et al.^26^ reported lower ∑_8_OPE concentrations, ranging from 0.35 to 8.40 ng L^–1^. The concentrations reported here are in the lower range of those reported in the South China Sea, lower than sites near the Amazon River, and comparable to those in the Pacific. These studies emphasize potential influences from regional sources and probably a number of environmental variables affecting inputs and biogeochemical cycling in the different oceanic regions. Surprisingly, the concentrations of OPE in the South Atlantic were not significantly different than those in the Southern Ocean, defying the expectation of lower concentrations in the latter due to its remote location.

**Figure 2:**
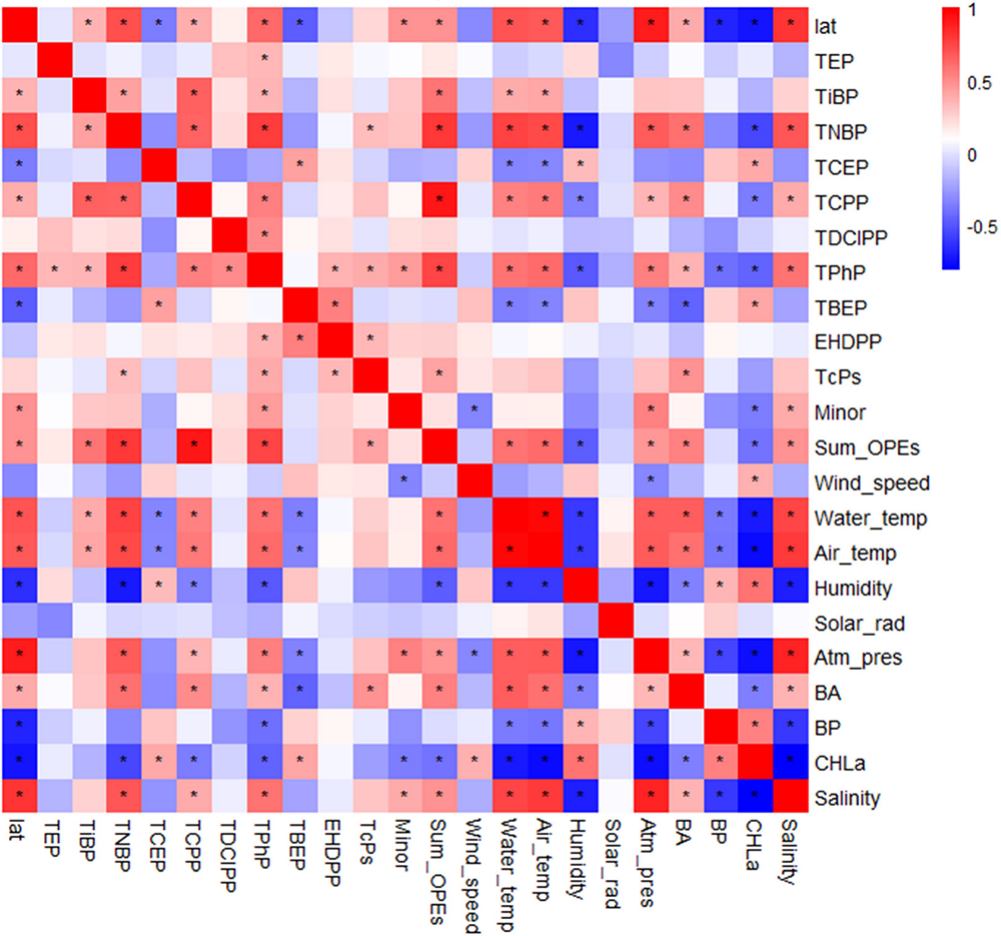
Spearman correlation heatmap showing the correlations between individual OPEs, ∑_24_OPE concentrations, and various biological (e.g., bacterial abundance, bacterial production, chlorophyll *a*) and environmental variables (e.g., water temperature, air temperature, and wind speed). Positive correlations are indicated in red and negative correlations are indicated in blue, with darker colors denoting stronger correlations. The asterisks indicate statistically significant correlations (*p* < 0.05).

Spearman correlation tests were conducted to assess the potential relationships between the OPE concentrations at 5 m depth and environmental and biological variables (Figure 2 and Table S11). Generally, individual OPEs were correlated among them, even though the relative abundance of individual OPEs was different in the Atlantic compared to the Southern Ocean. Bacterial abundance showed significant positive correlations with ∑_24_OPE, TCEP, ∑TCPP, ∑TcPs (Cl-OPEs), and minor OPEs, whereas it was negatively correlated with TBEP. This could indicate that certain bacteria might be tolerant to OPE toxicities, and although OPE background concentrations alone cannot explain the growth of some bacterial taxa, it suggests that OPEs can be used as a cometabolite growth substrate for some bacterial taxa along with other organic compounds. Recently, the biodegradation potential at six oceanic sites from the same north–south transect has been reported,^73^ showing that short-term (48 h) microbial degradation of OPEs, especially for hydrophobic aryl-OPEs, occurs when bacterial production is higher. This trend is consistent with the observed negative correlations between bacterial production and TPhP.

Chlorophyll *a* exhibited a negative correlation with ∑_24_OPE concentrations at 5 m depth, consistent with the biological pump removing OPEs from surface waters efficiently at sites with a larger phytoplanktonic biomass. Specifically, Chlorophyll *a* was negatively correlated with the surface concentrations of TPhP, TNBP, ∑TCPP, and the group minor-OPEs.

**Figure 3:**
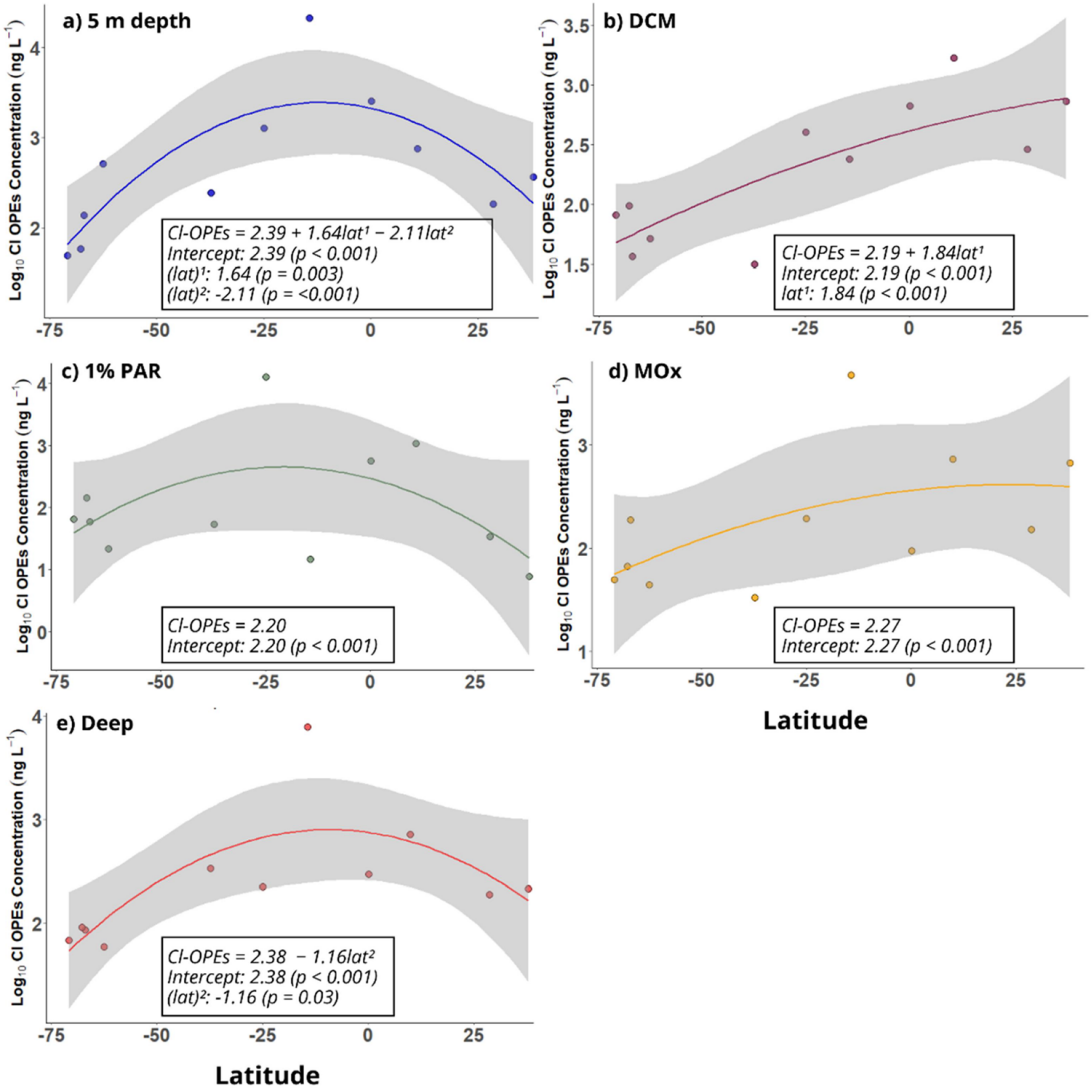
Latitudinal distribution of log Cl-OPE concentrations (ng L^–1^) at 5 m depth, at the deep chlorophyll maximum (DCM), at the depth with 1% of photosynthetically active radiation (1% PAR), at the minimum O_2_ depth (MOx), and at 2000 m depth (Deep). The significant variables (*p* < 0.05) of the quadratic correlations with latitudes are shown (Table S12).

Surface water and air temperatures were positively correlated to the concentrations of several OPEs, such as TPhP, ∑TCPP, TCEP, TNBP, and TiBP. To further explore these trends, a polynomial regression model was applied to log-transformed 5 m depth ∑_24_OPE concentrations against latitude, revealing a quadratic distribution (*R*^2^ = 0.39, *p* = 0.002) (Figures 3 and Table S11).

OPE increased from the northern hemisphere subtropical regions to the equatorial region, peaking at 14°S, and then decreased at higher southern latitudes, with the lowest concentrations in the Southern Ocean. This latitudinal trend at the surface was primarily due to the contribution of Cl-OPEs, confirmed with a significant quadratic correlation with latitude (*R*^2^ = 0.42, *p* < 0.001) (Table S12), but also observed for minor-OPES (*R*^2^ = 0.58, *p* < 0.001). Alkyl- and aryl-OPEs did not follow this trend at the surface, but in some cases with correlations with latitude at other depths (Table S12). This may be due to their lower persistence, and/or higher degradation and volatilization at high temperatures, as reported recently.^73^ At low latitudes, atmospheric and oceanic circulation close to the equator can bring OPEs from continents or proximate waters to this oceanic region. The decline of ∑_24_OPE, particularly Cl-OPE (and aryl-OPEs at the DCM depth), concentrations toward the Southern Ocean suggests a combination of reduced inputs and a potential influence of the biological pump’s removal of OPEs from surface waters (Figure 3), consistent with the inverse correlation between OPEs concentration and chlorophyll *a* reported above. Such a trend is more pronounced for Cl-OPEs than aryl-OPEs. Aryl-OPEs are generally more hydrophobic than Cl-OPEs and are biodegraded to a higher extent in warmer Atlantic waters than in the Southern Ocean.^73^ Still, at the DCM, aryl-OPEs decreased at the southernmost latitudes. Thus, the interpretation of latitudinal trends is complex for polar and biodegradable contaminants and does not follow a clear relationship with hydrophobicity as reported for legacy POPs.^74^

The higher concentrations observed in equatorial and intertropical regions are consistent with these sites being receptors of the prevailing oceanic and atmospheric currents, which may bring OPEs from South America and Africa, for instance, from the Amazon River-influenced region. Furthermore, this region is one of the few oceanic regions that receives atmospheric inputs from rain events.^75^

### Wet Deposition of OPEs

Wet deposition by rain is quantitatively important in some regions due to the high efficiency of raindrops scavenging air pollutants,^75−77^ leading to an amplification of concentrations in the receiving waters.^69^

The ∑_24_OPE concentrations in the two rain samples collected in, or proximate to, the Intertropical Convergence Zone (ITCZ), were 398.1 and 421.5 ng L^–1^ (averaging 409.9 ng L^–1^), more than 20-fold higher than 5 m depth ∑_24_OPE concentrations at the same stations (15.0 and 13.3 ng L^–1^, respectively) (Figure S2 and Table S13). ∑TCPP had a concentration of 270.4 and 288.5 ng L^–1^, accounting for 67.9 and 68.4% of ∑_24_OPE, respectively for the two rain events. In both samples, EHDPP (12.9 and 14.0%, respectively), and TiBP (6.07 and 8.84%, respectively) showed high contributions to ∑_24_OPE. The concentrations reported here for rain are 1 order of magnitude higher than the ones reported in Livingston Island (Antarctica),^69^ but in the lower range than those reported in Germany and Italy, where TCPP concentrations in rainwater ranged from 46 to 2659 ng L^–1^^78,79^ and from 28 and 739 ng L^–1^,^80^ respectively.

The wet deposition fluxes (*F*_WD_, ng m^–2^) are given by
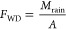
1where *M*_rain_ is the ∑_24_OPE mass in the rain (ng) and *A* (m^2^) is the collecting area (stainless steel funnel of 0.2 m diameter). The *F*_WD_ of ∑_24_OPE were 12,679 and 13,426 ng m^–2^ for the two rain events, respectively. The obtained F_WD_ implies that every rain event can increase the total OPE amount in surface waters by a few ng L^–1^. These large fluxes confirm that wet deposition can be a relevant source in the region and are consistent with the high concentration of Cl-OPEs found close to the equator. Such fluxes are the result of the large washout ratios or rain-air partition constant, (*K*_RP_), given by
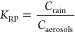
2where *C*_rain_ and *C*_aerosols_ are the concentrations in the rain (ng m^–3^) and aerosol (ng m^–3^) phase, respectively. We estimated *K*_RP_ using the *C*_rain_ measured here and *C*_aerosols_ reported for the Atlantic Ocean in a previous work^81^ (Table S14). K_RP_ ranged from 3.67 × 10^3^ ± 1.59 × 10^3^ (TEHP) to 1.39 × 10^6^ ± 8.27 × 10^4^ (TPhP) for individual OPEs. Log *K*_RP_ ranged from 3.5 (TEHP) to 6.1 (TPhP) with an average of 4.9 (Table S15). There were no significant correlations between *K*_RP_ and the hydrophobicity of the OPE’s hydrophobicity. These *K*_RP_ are of similar magnitude to those reported before, which ranged from 4.1 to 7.3.^69^ Nevertheless, the rain concentrations could have a contribution from gas-phase OPEs, which are not available in these estimates. Wet deposition has been identified as a dominant mechanism for the deposition of aerosol-bound pollutants in the Atlantic Ocean,^75^ particularly in regions with high precipitation rates, such as the Intertropical Convergence Zone (ITCZ), where our samples were collected. Both the general circulation pattern of air masses and river inputs and the importance of wet deposition could contribute to the high OPE concentrations at low latitudes.

### OPEs in the Top Meters of the Ocean

Generally, surface-named concentrations refer to seawater taken at 4–5 m depth but not the real surface of the ocean, which cannot be sampled directly from an oceanographic ship. Recently, the concurrent OPE concentrations in the surface microlayer (SML) and subsurface layer (SSL) have been reported at 18 stations from the same transect.^54^ This allows one to compare the OPE concentrations at the sea skin (SML, top mm of the ocean), at 0.5 m depth (SSL), and at 5 m depth, providing insights into the dynamics of contaminants in the top meters of the ocean.

The surface microlayer is the interface between the atmosphere and the ocean, potentially concentrating OPEs due to their physicochemical properties, whereas the top meters of the ocean are continuously mixed by convection and turbulence driven by temperature and wind. In the field, the SML can be collected from an inflatable boat using a glass plate sampler.^54^ The comparison of the OPE concentrations at SML, SSL, and 5 m depth is done for 18 stations, nine stations at each ocean, where the three depths were sampled concurrently (Figure S3). In the Atlantic Ocean, the 5 m depth, SSL, and SML average ± SD concentrations (ng L^–1^) were 25.7 ± 22.4, 12.6 ± 7.54, and 24.7 ± 16.0, respectively. In the Southern Ocean, the 5 m depth, SSL, and SML average ± SD concentrations (ng L^–1^) were 7.18 ± 3.42, 16.5 ± 9.17, and 193.0 ± 143.0, respectively. For the Atlantic Ocean, there were no significant ∑_24_OPE concentration differences among the layers (Kruskal–Wallis, *p* = 0.09). Pairwise Wilcoxon tests revealed no significant differences between 5 m and SML (*p* = 0.93) or between 5 m and SSL (*p* = 0.08). However, the difference between SML and SSL was significant (*p* = 0.05), consistent with the enrichment factors generally higher than unity between the SML and SSL.^54^

In contrast, the Southern Ocean’s concentrations showed significant differences between the 5 m depth and SML (*p* = 0.0001), the 5 m depth and SSL (*p* = 0.01), and the SML and SSL (*p* = 0.003). These results show an important gradient of OPEs in the top 5 m of the water column, which may be facilitated by gradients of salinity (from ice and snow melting), temperature, or higher biological activity leading to exudates of surfactant-like substances in the Southern Ocean than in the Atlantic Ocean. Conversely, in tropical and subtropical waters, the top 5 m shows moderately constant concentrations of the OPEs, possibly related to temperature facilitating either degradation or volatilization of the OPEs close to the surface interface, to a larger extent than at 5 m.

### OPE in the Phytoplankton Maximum (DCM Depth)

DCM depths ranged from 34 to 145 m in the Atlantic and from 19 to 57 m in the Southern Ocean. In the North Atlantic, ∑_24_OPE mean concentrations at the DCM were 19.3 ng L^–1^, not significantly different than at 5 m depth (mean 18.6 ng L^–1^ for the same stations), and neither for any individual OPE. However, in the South Atlantic, 5 m depth concentrations, averaging 27.6 ng L^–1^, were significantly higher (*p* < 0.05) than in the DCM, with an average of 14.5 ng L^–1^ for ∑_24_OPE, and also significantly different for TNBP, TCEP, TDCIPP, and EHDPP.

**Figure 4:**
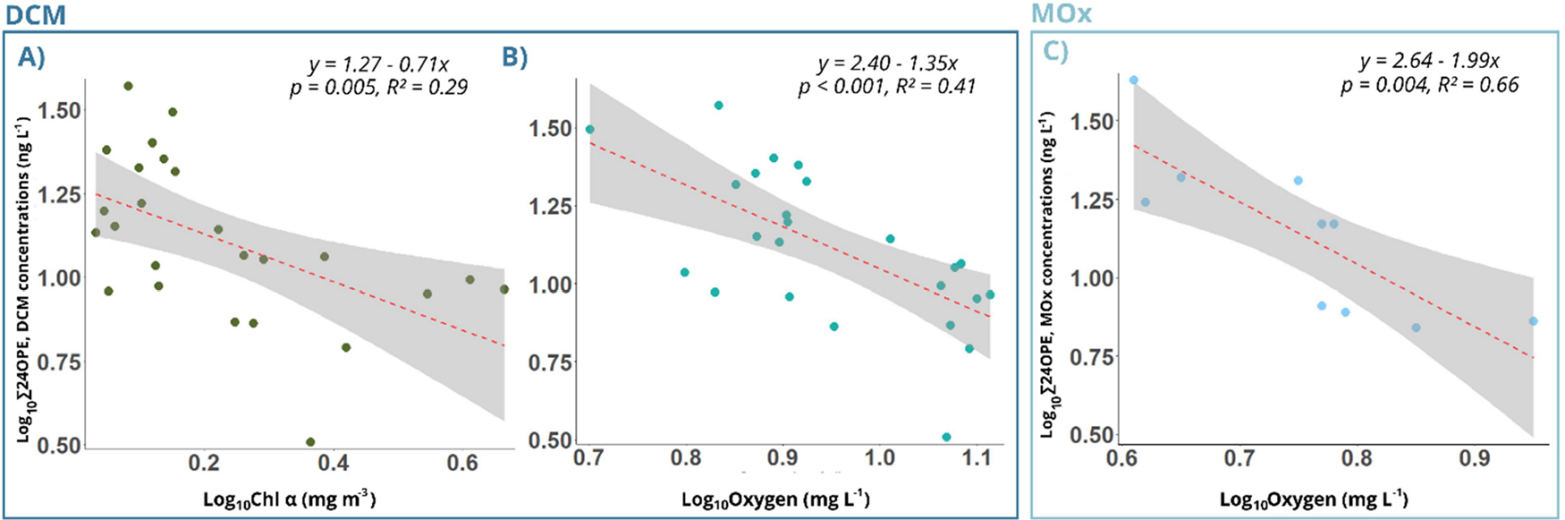
Linear regressions between Σ_24_OPE concentrations (ng L^–1^) and Chl *a* (mg m^–3^) (panel A), and oxygen levels (mg L^–1^) at the DCM (panel B). Panel C shows the linear regression between Σ_24_OPE concentrations (ng L^–1^) and oxygen levels (mg L^–1^) at the MOx layer (panel C). Red dashed lines represent the linear regression, and the shaded areas indicate the 95% confidence intervals. Linear regression equations and *p*-values are shown for each plot.

A significant positive correlation between latitude and log ∑_24_OPE concentrations at the DCM (*p* = 0.002, *R*^2^ = 0.34) was observed, with the highest concentrations in the northern hemisphere decreasing steadily toward southern latitudes, with minimum concentrations in the Southern Ocean. This pattern was also significant for Cl-OPEs (*R*^2^ = 0.36, *p* = 0.001) (Figure 3), aryl-OPEs (*R*^2^ = 0.41, *p* = 0.001), and minor-OPEs (*R*^2^ = 0.36, *p* = 0.001). Such a latitudinal trend could be related to the fact that the sampling was performed in different seasons, winter for the northern hemisphere samples (December 2020), and summer for the southern hemisphere samples (January 2020 and January–February 2022). This implies samples from different stages of seasonal physical and biological succession. There were significant negative correlations between Chl *a* (mg m^–3^) and ∑_24_OPE concentrations (*r* = −0.54, *p* = 0.005) and between water oxygen (mg L^–1^) and ∑_24_OPE concentrations (*r* = −0.64, *p* = 0.0005) (Figure 4). This trend is consistent with the negative correlation of the OPEs and Chl *a* at 5 m depth reported above (Figure 2). In the northern hemisphere, during the winter season sampling, a lower phytoplankton biomass and a weakened biological pump likely reduced the level of removal of OPE, resulting in higher concentrations of OPE at the DCM. In contrast, the lower concentrations of OPE observed at lower latitudes (Southern and South Atlantic oceans) are consistent with an enhanced biological pump when biomass was higher (evidenced by higher Chl *a*). Similar trends have been observed for other pollutants, such as PFAS,^19^ which also tend to decrease from the surface to the DCM. It is also possible that higher bacterial abundances lead to increased degradation, though this may occur more slowly in colder waters. While some individual OPEs have been shown to serve as a phosphorus source,^67^ phosphorus is generally not a limiting nutrient in the Southern Ocean. On the other hand, microbial degradation of OPEs due to carbon demand has been suggested to be minimal in the Southern Ocean.^73^

### OPEs in the Mesopelagic and Deep Ocean

**Figure 5:**
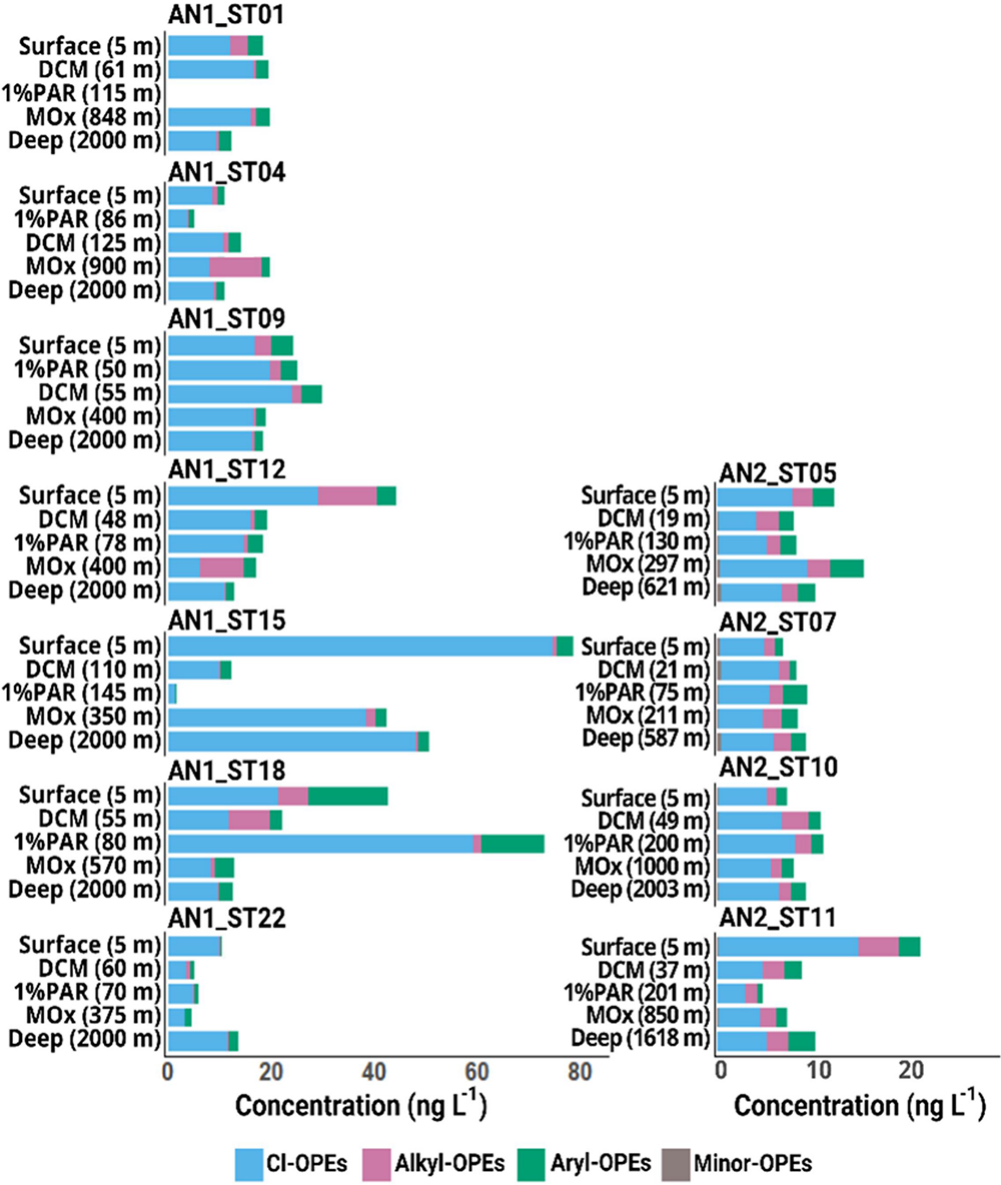
Vertical profiles of concentrations (ng L^–1^) of grouped OPEs categorized as alkyl-OPEs, aryl-OPEs, Cl-OPEs, and minor OPEs. The left panels show the vertical profiles sampled in the Atlantic Ocean, whereas the right panels show profiles sampled in the Southern Ocean. Please note that the *x*-axis in the left and right panels are not shown at the same scale.

Few studies have reported OPEs at water layers below the photic zone. In addition to 5 m and DCM depths, OPEs were measured at the depth with the 1% photosynthetically active radiation (1%PAR), the oxygen minimum layer (MOx), and at 2000 m depth at 11 stations (Tables S16–S18). In both the Atlantic and Southern Oceans, Cl-OPEs consistently accounted for the largest contribution to ∑_24_OPE at all depths, but this contribution was lower in the Southern Ocean (Tables S17 and S18). Overall, OPE concentrations did not exhibit consistent trends with depth, as some stations showed a surface enrichment with a depth depletion profile (station AN1_ST12) consistent with sources at the surface and vertical transport due to the biological pump. Other depth profiles displayed both surface enrichment and an increase in concentrations with depth (station AN1_ST15), while some profiles showed no discernible pattern (Figures 5 and S5). Overall, the biogeochemistry of polar, potentially biodegradable chemicals such as OPEs follows complex processes, following less prominent trends with depth than when evaluated latitudinally for a single depth (Figure 3).

Cl-OPEs, alkyl-OPEs, and aryl-OPEs at the 1%PAR depth showed no significant correlations with latitude (Figure 3 and Table S12), even though some decrease in concentrations in the Southern Ocean and Northern Hemisphere concentrations was observed. This contrasts with the latitudinal trends at 5 m and DCM depths discussed above. At 1% PAR, the lower boundary of the euphotic zone, phytoplankton photosynthesis can still occur but at minimal rates. Thus, depletion of dissolved-phase OPEs by the biological pump is likely to be minimal, with the latitudinal profile at this depth more closely resembling that observed at 5 m (source-influenced) than at the DCM (influenced by the biological pump).

From the concentrations at the surface, the DCM and 1%PAR, and the volumes of the oceanic basins,^82^ a reservoir of 119, 190, and 273 Ktons of ∑_24_OPE were estimated for the North Atlantic, South Atlantic, and Southern Ocean, respectively. This reservoir is comparable to a lower-end estimate of the global annual production of OPEs of 598 Ktons.^83^ The reservoir in the deep ocean cannot be estimated because it would require a larger sampling resolution.

The MOx depth has received increasing attention during the past decade as a relevant biogeochemical layer,^84,85^ even though it has not yet been explored in terms of biogeochemical cycling of contaminants. At the MOx layer, oxygen levels fall below 80 μmol kg^–1^, which limits oxygen-sensitive species, thus altering biogeochemical processes.^86^ In addition, microanaerobic environments in marine snow aggregates may be feasible. Such low levels of O_2_ can originate from remineralization (respiration) of sinking organic matter (OM). Such depletion of OM would induce a redissolution of OPEs due to particle–water partitioning since when the OM decreases, there is a fugacity amplification of OPEs due to solvent depletion. A significant positive latitudinal trend for log-∑_24_OPE was observed (*R*^2^ = 0.48, *p* = 0.03), indicating higher OPE concentrations in the northern hemisphere, and decreasing concentrations at the southernmost latitudes. A significant influence of latitude at the MOx depth was also found for minor OPES.

Additionally, a significant negative correlation between ∑_24_OPE concentrations at MOx and oxygen levels (*r* = −0.81, *p* = 0.004) was found (Figure 4), suggesting higher OPE concentrations in oxygen-depleted regions. The latitudinal trend at the MOx layer is similar to that found at the DCM but different than at the surface and 1%PAR. This is consistent with the OPE concentrations, reflecting the extent of the biological pump, with a fraction of the exported OPEs redissolved due to amplified fugacity and particle–water partitioning at the MOx layer. Nevertheless, further research is needed on the biogeochemistry of contaminants in O_2_-depleted zones, which are increasing under the current scenario of global change.^85^

The deep-water samples (2000 m depth) provide a long-term perspective of the accumulation of the OPEs in the deep ocean. This layer integrates the signal over long time periods, as many biogeochemical processes are slow at this depth. Therefore, it reflects cumulative biogeochemical processes and OPEs received from overlying waters over time, even though advection of deep water masses occurs, and this may lead to spatial changes in contaminant accumulation, variability, and differences between the geographic distribution at the surface and deep waters. A significant quadratic relationship (*p* = 0.03) was observed for Cl-OPE concentrations in deep waters, analogous to that observed in surface waters, following an inverted U-shaped distribution across latitudes (Figure 3). Additionally, a significant positive correlation was found between surface and deep concentrations for Cl-OPEs (*r* = 0.68, *p* = 0.03), aryl-OPEs (*r* = 0.63, *p* = 0.04), and minor-OPEs (*r* = 0.61, *p* = 0.04), suggesting that as surface concentrations reflect sources, deep waters integrate these sources over time, showing a similar latitudinal trend (Figure S6). No significant correlations were observed for alkyl-OPEs, indicating different transport and degradation mechanisms. OPE at the MOx depth was also correlated with those at the 2000 m depth (*r* = 0.76, *p* = 0.006), suggesting that deep waters reflect not only the OPE sources at the surface but also different vertical transport and biogeochemical mechanisms that impact the concentrations of the OPE across multiple layers.

OPE throughout the water column suggests that the surface layer, representing the primary and secondary sources of OPEs, plays a dominant role. The MOx layer, similar to DCM but influenced by surface processes, serves as a transitional zone. The biological pump plays a crucial role in transporting vertically OPEs attached to sinking organic matter, which when remineralized at deeper waters, can liberate OPEs back to the dissolved phase. This results in a mixed pattern in MOx and 1%PAR layers but a clearer pattern in deep waters, which integrate sources and biogeochemistry, where the OPE concentrations align with surface distributions. Over time, the biological pump removes OPEs from the surface and transports them to deeper layers, with microbial degradation probably influencing OPE distribution at intermediate depths, the elucidation of which will require future work. Research priorities should focus on the elucidation of the seasonal variability at different oceanic regions as well as a more detailed assessment of vertical distributions and how particle-seawater partitioning varies with depth. The large latitudinal sampling effort performed here has proven useful in discerning patterns and the processes affecting the OPEs in the Ocean and shows that the OPEs are mobile and persistent enough to reach the whole ocean.

## Supporting Information Available

Additional text on methods (note S1), additional figures showing concentrations and correlations (Figures S1–S7), additional tables with ancillary data, quality assurance, target compounds information, individual OPEs concentrations, washout ratios, and correlations (Tables S1–S18) (PDF)
