# Supplementary material for: Biogeochemical Controls on Latitudinal (42°N to 70°S) and Depth Distribution of Organophosphate Esters in the Atlantic and Southern Oceans
Source: Environ Sci Technol. 2025 Mar 11;59(11):5585–95. doi: 10.1021/acs.est.4c12555 (PMC11948325; doi:10.1021/acs.est.4c12555)
Supplement: Supplementary file 1 — es4c12555_si_001.pdf [file es4c12555_si_001.pdf]

Supplementary Material

Biogeochemical Controls on Latitudinal (42°N to 70°S) and Depth Distribution of Organophosphate Esters in the Atlantic and Southern Oceans

Núria Trilla-Prieto[1,2], Naiara Berrojalbiz[1], Jon Iriarte[1], Antonio Fuentes-Lema[3], Cristina Sobrino[4], Maria Vila-Costa[1], Begoña Jiménez[5], and Jordi Dachs[1]*

[1] Department of Environmental Chemistry, IDAEA-CSIC, 08034, Barcelona, Catalunya, Spain.

[2]Departament of Ecology, Faculty of Earth Sciences, Universitat de Barcelona, 08034, Barcelona, Catalunya, Spain.

[3]Centro de Investigación Mariña (CIM), Universidade de Vigo, 36310, Vigo, Spain

[4]Grupo de Oceanografía Biolóxica, Centro de Investigación Mariña (CIM), Universidade de Vigo, 36310, Vigo, Spain.

[5]Department of Instrumental Analysis and Environmental Chemistry, IQOG-CSIC, 28006, Madrid, Spain.

*Corresponding Author: jordi.dachs@idaea.csic.es.

This supplementary material contains 29 pages with 1 note, 7 figures and 18 tables.

Summary

Notes

Note S1. Analytical methodologies for Chlorophyll *a*, bacterial production (BP) and bacterial abundance (BA) measuring from seawater samples.

Figures:

Figure S1. Log-transformed concentrations of grouped OPEs vs latitude, colored by depth (m). Panel top left plots Cl-OPES, top right Alkyl-OPEs, bottom left Aryl-OPEs and bottom right Minor-OPEs. The color gradient represents the sampling depth, with shallower depths depicted in yellow and deeper depths in purple. The shaded area around the regression line represents the 95% confidence interval.
Figure S2. Rainfall and 5 m depth seawater (Surface) individual and $\sum_{24}$OPE concentrations (ng L^{-1}) from Atlantic Ocean stations AN1_ST10 and AN1_ST11.
Figure S3 Surface microlayer (SML), subsurface layer (SSL), and 5m depth OPE concentrations (ng L^{-1}) of individual and $\sum_{24}$OPE at nine stations sampled in the Atlantic Ocean (top panel) and the Southern Ocean (bottom panel).
Figure S4. Scatter plots comparing the individual and $\sum_{24}$OPE log-transformed concentrations between the 5 m depth (Surface) and DCM layers across three ocean regions.
Figure S5. Vertical profiles of concentrations (in ng L^{-1}) of $\sum_{24}$OPEs plotted over the bathymetry of the sampling site where each vertical profile was sampled. T
Figure S6. Log-transformed surface (5 m depth) and deep (2000 m) OPE concentrations for different OPE groups (Cl-OPEs, Alkyl-OPEs, Aryl-OPEs, and Minor OPEs). Spearman correlation coefficients (r) and p-values are displayed for each group.
Figure S7. Major ocean gyres (top) and global winds circulation (bottom).

Tables

Table S1. Stations information from the Atlantic Ocean (top table) and Southern Ocean (bottom table) where samples for OPE analysis were collected.
Table S2. Information of labelled OPE compounds used as surrogate (top table) and as Internal Standard (bottom table).
Table S3. Names, acronym molecular weight (MW) and Log KOW of the targeted compounds.
Table S4. Individual OPE contribution (%) to the total pool for each for the Atlantic and Southern Ocean samples.
Table S5. Field and procedural blanks OPE abundance (ng).
Table S6. Limits of quantification (LOQs) in ng.
Table S7. Surrogate and matrix spike recoveries (%) from the Atlantic Ocean samples (top table) and Southern Ocean samples (bottom table).
Table S8. Individual and $\sum_{24}$OPE concentration in surface and DCM samples from the Atlantic Ocean (ng L^{-1}).
Table S9. Mean and SD of surface and DCM concentrations (ng L^{-1}) in the three ocean basins sampled.
Table S10. Individual and $\sum_{24}$OPE concentration in surface and DCM samples from the Southern Ocean (ng L^{-1}).
Table S11. Environmental variables measured at surface waters and atmospheric conditions in the Atlantic and Southern Oceans.

Table S12. Summary of the polynomial regression models applied to log-transformed grouped OPEs concentrations across different depths (5 m, DCM, 1% PAR, MOx, and Deep) with latitude.
Table S13. Wet Deposition and related surface sample concentrations (in ng L^{-1}) of individual and ∑24OPE in the Atlantic Ocean and calculation of EF_{WD} (C_{WD}/C_{SURF}) for those samples.
Table S14. Atmospheric aerosol concentrations of $\sum_{14}$OPEs (ng m^{-3}) from Malaspina (Castro-Jiménez et al., 2016) from latitudes ranging between 7°N and 0°N (top table) and rain water $\sum_{14}$OPEs (ng m^{-3}) concentrations from AN1_ST10 and AN1_ST11 2021 Atlantic campaign.
Table S15. Log K_{RP} values from the Atlantic Ocean.
Table S16. Mean ± SD of group OPE concentrations (ng L^{-1}) separated by ocean and depth.
Table S17. Individual and $\sum_{24}$OPE concentration in vertical profiles from the Atlantic Ocean (ng L^{-1}).
Table S18. Individual and $\sum_{24}$OPE concentration in vertical profiles from the Southern Ocean (ng L^{-1}).

Note S1. Analytical methodologies for chlorophyll *a*, bacterial production and bacterial abundance measuring from seawater samples.

Chlorophyll *a* (Chl *a*) concentrations were measured in all samples. For each sample, 250 mL was gently filtered under dim light in triplicate through a size fraction cascade system with 20, 2 and 0.22 µm size pore 47-mm Filter-Lab polycarbonate filters and immediately stored at -20 °C until further analysis. Chl a extraction was carried out with 90% acetone, and the concentration in each sample (µg L^{-1}) was estimated following the acidification method described by Estrada (2012) using a Turner A10 fluorometer previously calibrated with a pure Chl *a* standard (SIGMA C5753-1 mg). Here, we provided the sum of the three fractions as a proxy for biomass and biological activity.

Bacterial Production (BP) (as Leucine Incorporation Rates (LIR)) was estimated as the incorporation of 3H-leucine into protein, as reported previously.[2] Briefly, 1.2 mL quadruplicate live and duplicate killed (5% trichloroacetic acid (TCA)) subsamples were incubated with 3H-leucine (40 nM) for 4-5 hours at in situ temperature in the dark. Incubation was stopped by the addition of 120 µL of cold TCA 50% and then frozen (−20 °C) until further processing by centrifugation and TCA rinsing.

Prokaryotic cell abundance (Bacterial Abundance, BA) was determined using flow cytometry. Subsamples of 1.8 mL for quantification of prokaryote abundance were fixed with 1% buffered paraformaldehyde solution (pH 7.0) plus 0.05% glutaraldehyde, left at room temperature in the dark for 10 min, flash-frozen in N_2 (l), and stored at −80 °C. Prokaryotic cell abundance was estimated using flow cytometry, as described elsewhere [3]. Briefly, the samples were run using a BD Accuri C6 Plus flow cytometer. The resulting cytograms were analyzed using FlowJo software to quantify the high-nucleic acid-containing (HNA) and the low-nucleic acid-containing (LNA) prokaryotes.

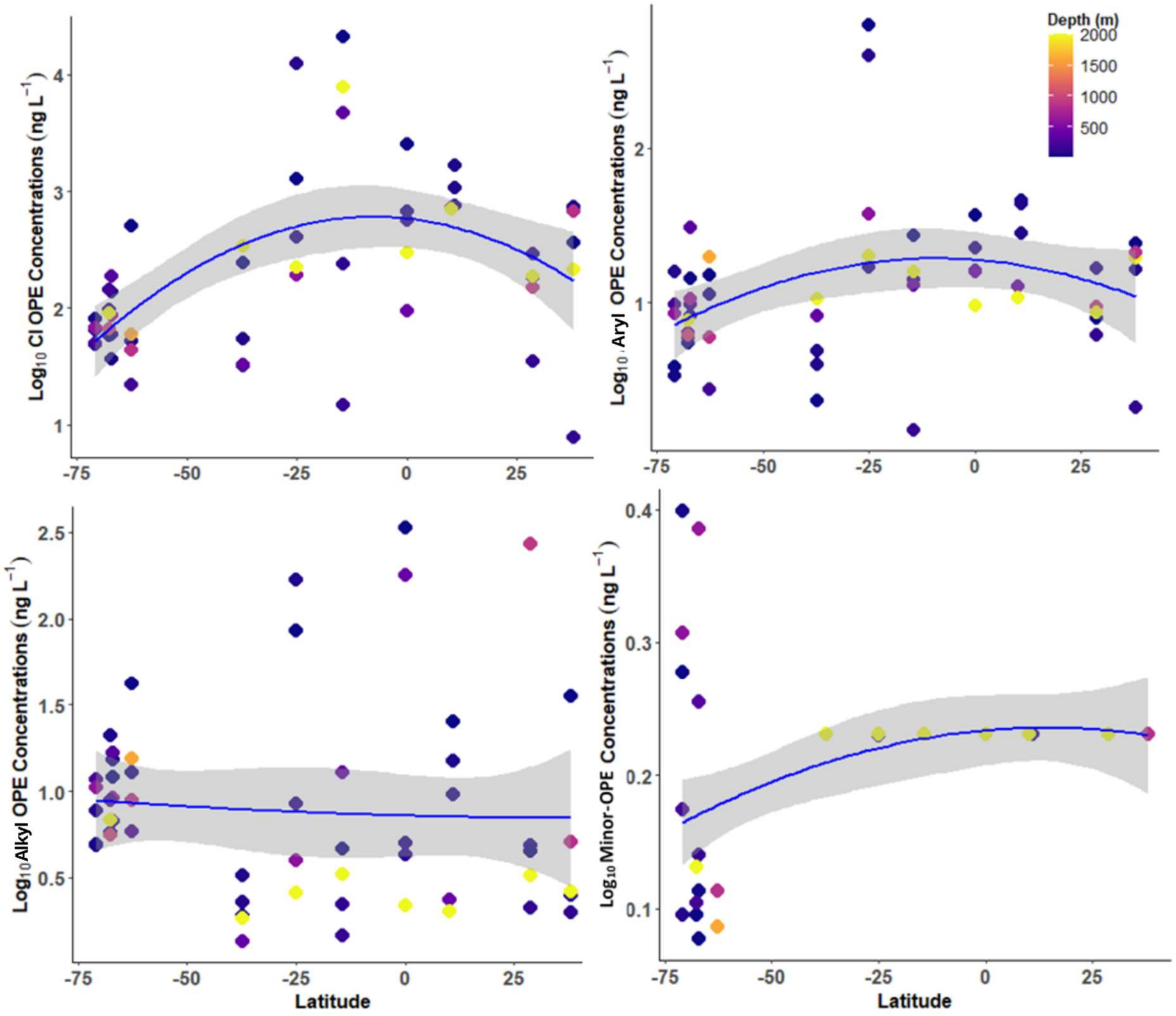

Figure S1. Log-transformed concentrations of grouped OPEs vs latitude, colored by depth (m). Panel top left plots Cl-OPES, top right Alkyl-OPEs, bottom left Aryl-OPEs and bottom right Minor-OPEs. The color gradient represents the sampling depth, with shallower depths depicted in purple and deeper depths in yellow. The shaded area around the regression line represents the 95% confidence interval.

Statistical results for polynomial regression model considering all samples (all depth. See Table S12 for correlations at each depth):

- **Cl-OPEs** show a significant, non-linear relationship with latitude ($p < 0.001$, $R^2 = 0.34$).
- **Aryl-OPEs** show a non-linear influence of latitude ($p < 0.001$, $R^2 = 0.16$).
- **Alkyl-OPEs** show no significant effects from latitude**.**
- **Minor-OPEs** show a significant positive linear relationship with latitude ($p < 0.001$, $R^2 = 0.58$).

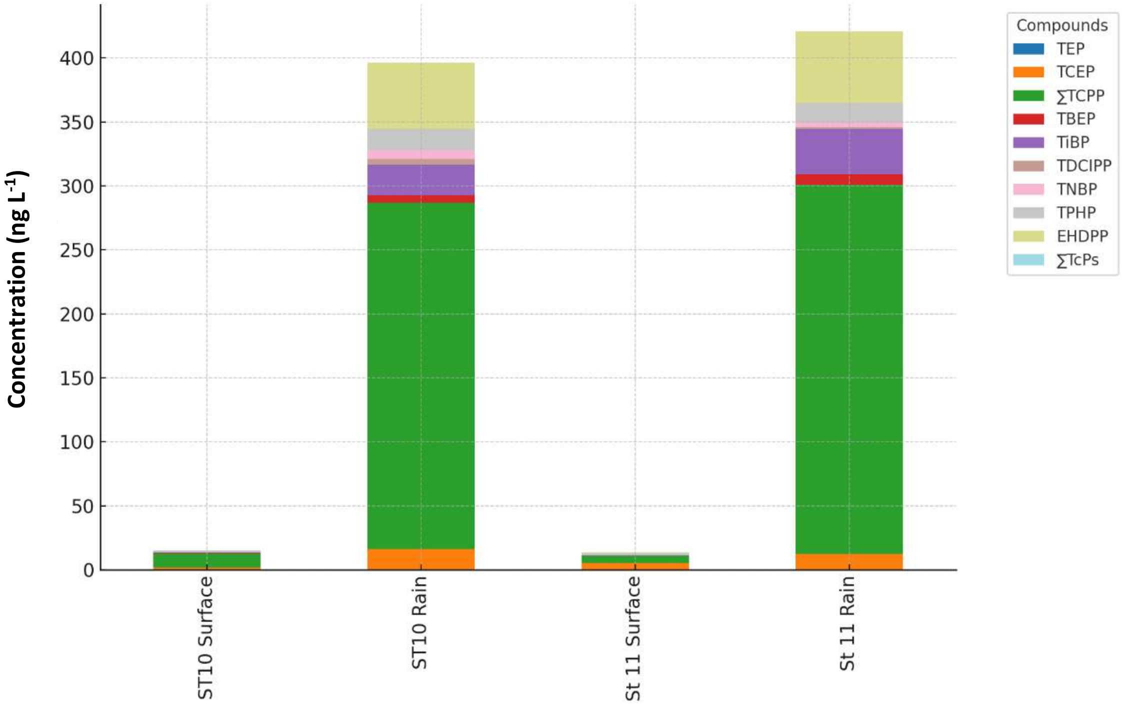

Figure S2. Rainfall and 5 m depth seawater (Surface) individual and $\sum_{24}$OPE concentrations (ng L^{-1}) from Atlantic Ocean stations AN1_ST10 and AN1_ST11.

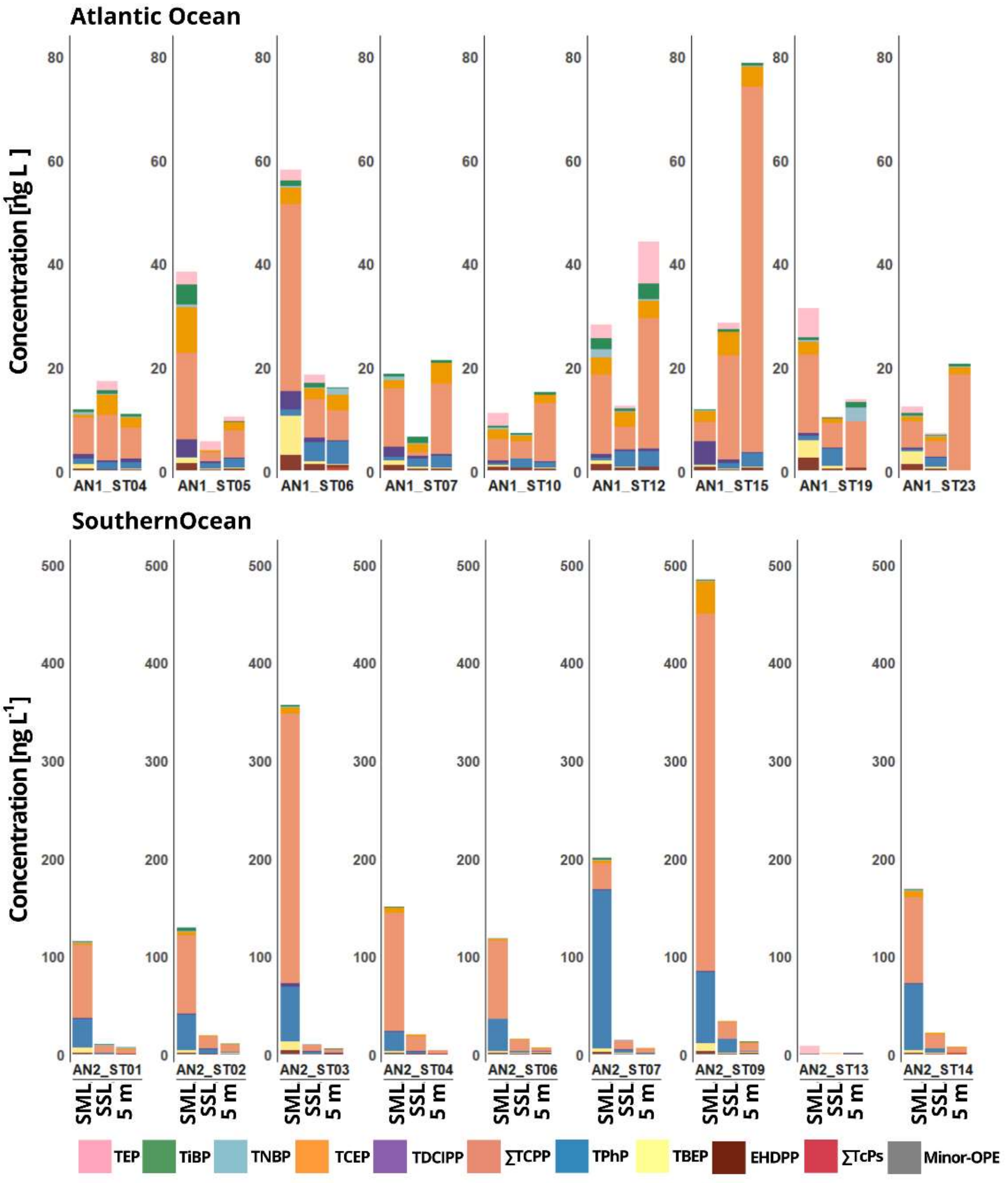

Figure S3 Surface microlayer (SML), subsurface layer (SSL), and 5m depth OPE concentrations (ng L^{-1}) of individual and $\sum_{24}$OPE at nine stations sampled in the Atlantic Ocean (top panel) and the Southern Ocean (bottom panel).

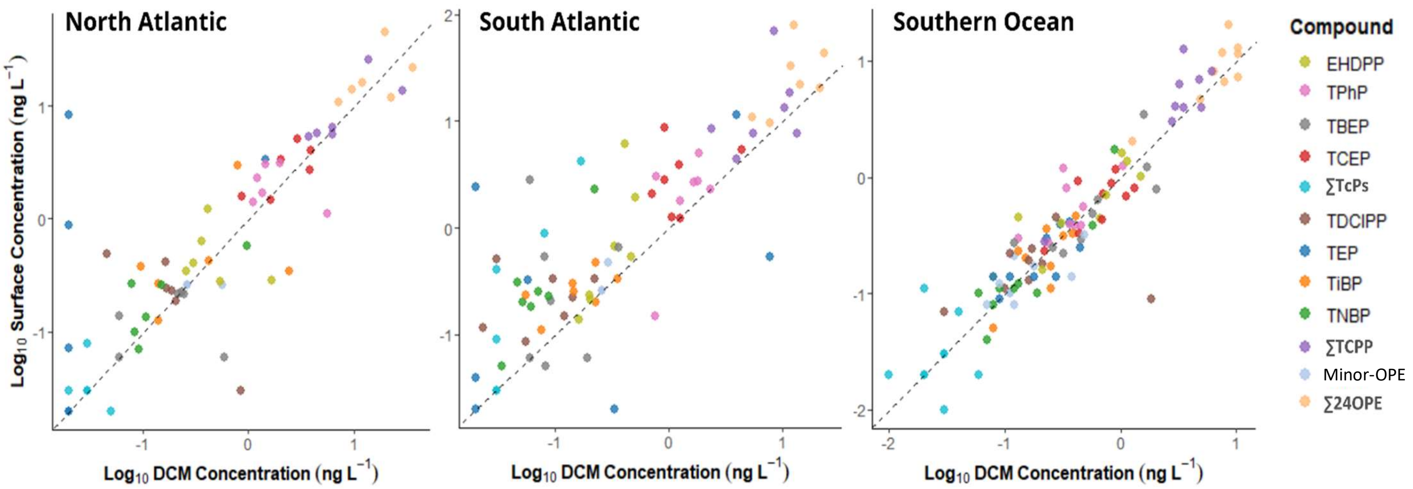

Figure S4. OPEs in surface versus DCM. Scatter plots comparing the individual and $\sum_{24}$OPE log-transformed concentrations between the 5 m depth (Surface) and DCM layers across three ocean regions: North Atlantic, South Atlantic, and Southern Ocean. The dashed line represents a 1:1 relationship between surface and DCM concentrations.

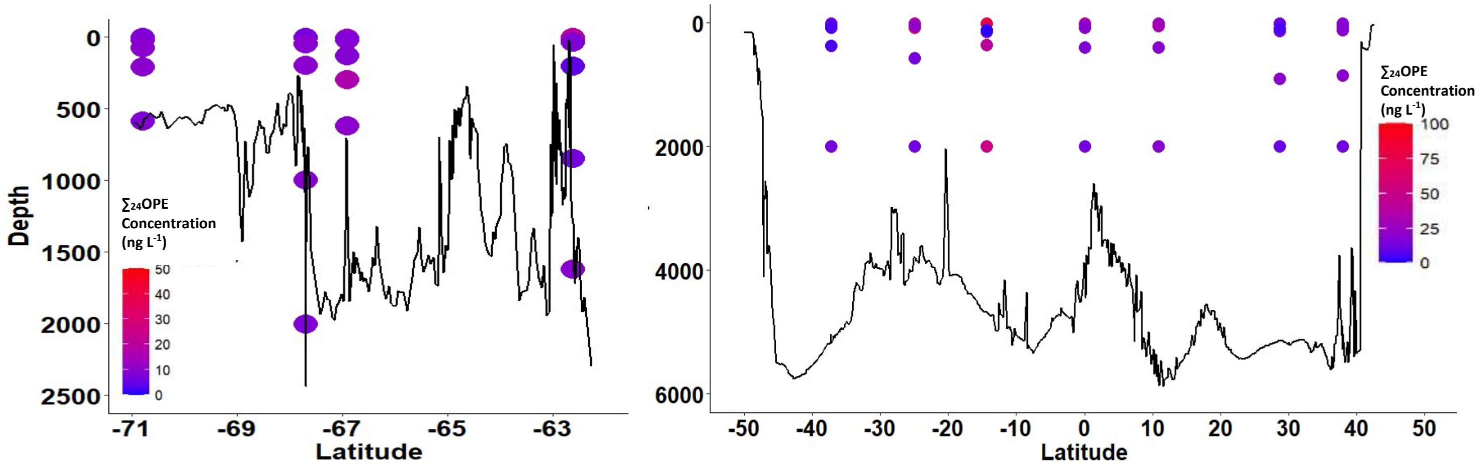

Figure S5. Vertical profiles of $\sum_{24}$OPE concentrations (in ng L^{-1}) plotted over the bathymetry during the transects for the ANTOM-1 and ANTOM-2 cruises. The left panel shows vertical profiles of concentrations sampled in the Southern Ocean (63°S to 71°S) and the right panel shows vertical profiles of concentrations sampled in the Atlantic Ocean (40°N to 40°S).

Figure S6. Log-transformed surface (5 m depth) versus deep (2000 m) OPE concentrations for different OPE groups (Cl-OPEs, Alkyl-OPEs, Aryl-OPEs, and Minor-OPEs). Spearman correlation coefficients (r) and p-values are displayed for each group.

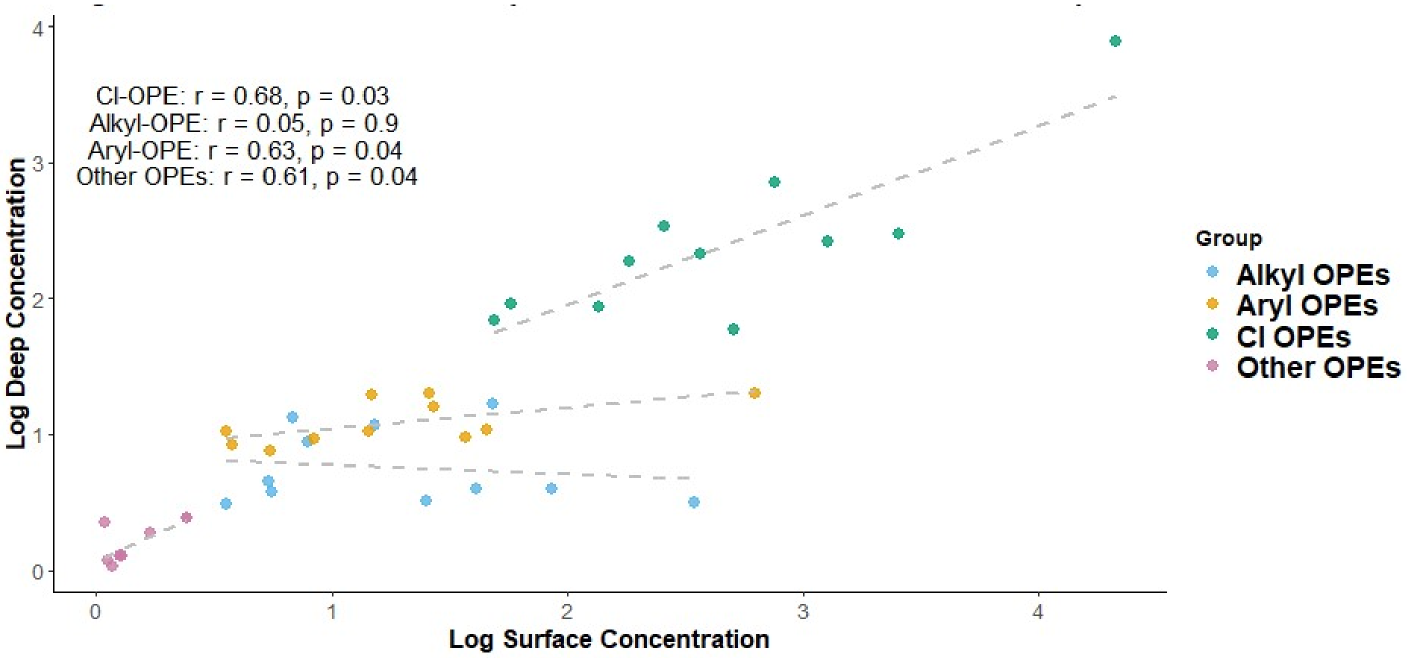

Cl-OPEs, aryl-OPEs, and minor-OPEs show significant positive correlations between surface and deep concentrations (r = 0.68, p = 0.03, r = 0.63, p = 0.04, and r = 0.61, p = 0.04, respectively), while alkyl-OPEs exhibit weaker or no significant correlation.

Figure S7. Major ocean gyres and currents.

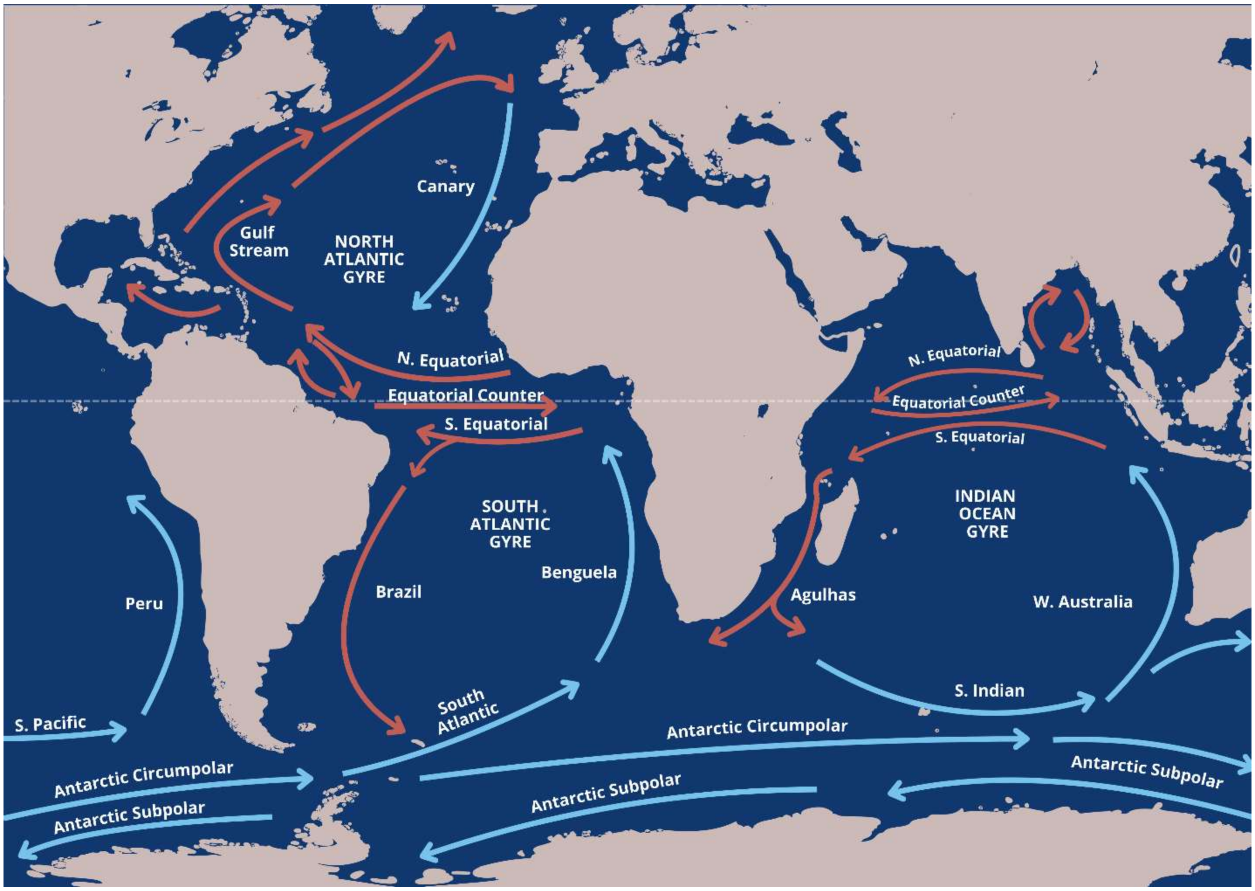

Table S1. List of samples, date and coordinates from the Atlantic Ocean (top table) and Southern Ocean (bottom table) where samples for OPE analysis were collected.

Station Code	Date	Latitude	Longitude	Surface sample	DCM sample	Vertical Profile	WD Sample
AN1_St01	18/12/2020	37.91	-15.97	x	x	x	
AN1_St02	19/12/2020	35.73	-19.06	x	x		
AN1_St03	20/12/2020	32.18	-21.53	x	x		
AN1_St04	21/12/2020	28.65	-23.39	x	x	x	
AN1_St05	22/12/2020	25.63	-24.89	x	x		
AN1_St06	23/12/2020	22.22	-26.63	x			
AN1_St07	24/12/2020	18.86	-28.31	x	x		
AN1_St08	25/12/2020	14.98	-28.77	x			
AN1_St09	26/12/2020	10.87	-29.23	x	x	x	
AN1_St10	27/12/2020	7.34	-29.58	x			x
AN1_St11	28/12/2020	3.82	-29.86	x	x		x
AN1_St12	29/12/2020	0.15	-30.50	x	x	x	
AN1_St13	30/12/2020	-2.97	-30.95	x			
AN1_St14	31/12/2020	-6.77	-31.33	x	x		
AN1_St15	02/01/2021	-14.36	-33.91	x	x	x	
AN1_St16	03/01/2021	-17.77	-35.26	x			
AN1_St17	04/01/2021	-21.29	-36.81	x	x		
AN1_St18	05/01/2021	-24.91	-38.71	x	x	x	
AN1_St19	06/01/2021	-27.79	-40.65	x			
AN1_St20	07/01/2021	-30.98	-42.96	x	x		
AN1_St21	08/01/2021	-34.40	-45.63	x			
AN1_St22	09/01/2021	-37.24	-48.09	x	x	x	
AN1_St23	11/01/2021	-43.79	-54.75	x			
AN1_St24	12/01/2021	-46.78	-58.46	x	x	x	

Station code	Date	Latitude	Longitude	Surface sample	DCM sample	Vertical Profile
AN2_St01	23/01/2022	-62.71	-60.64	X	X	
AN2_St02	24/01/2022	-62.92	-61.04	X	X	
AN2_St03	25/01/2022	-64.58	-62.57	X		
AN2_St04	26/01/2022	-65.15	-65.64	X	X	
AN2_St05	27/01/2022	-66.92	-69.10	X	X	X
AN2_St06	28/01/2022	-67.75	-68.03	X		
AN2_St07	30/01/2022	-70.85	-79.89	X	X	X
AN2_St08	30/01/2022	-70.85	-79.89	X		
AN2_St09	31/01/2022	-69.21	-78.84	X	X	
AN2_St10	01/02/2022	-67.70	-75.00	X	X	X
AN2_St11	04/02/2022	-62.57	-58.62	X	X	X
AN2_St13	05/02/2022	-62.97	-60.29	X	X	
AN2_St14	06/02/2022	-62.95	-60.64	X	X	

Table S2. Information of labelled OPEs used as surrogate (top table) and as Internal Standard (bottom table).

**Compound name**	**Abbreviation**	**Concentration (ng µL^{-1})**	**V added (µL)**	**Mass added (ng)**
Tri-n-buthyl phosphate - d27	TNBP-d27	1	50	50
Triphenyl phosphate - d15	TPhP-d15	1	50	50
Tris (2-chloroethyl) phosphate - d12	TCEP-d12	1	50	50
Tri-n-propyl phosphate - d21	TPrP-d21	1	50	50
Tris (2-chloroisoprpyl) phosphate - d18	TCPP-d18	1	50	50
Tris (2-ethylhexyl) phosphate - d51	TEHP-d51	1	50	50
Triphenylphosphine oxide - d15	TPPO-d15	1	50	50

**Compound name**	**Abbreviation**	**Concentration (ng µL^{-1})**	**V added (µL)**	**Mass added (ng)**
Tris (1.3-dichloro-2-propyl) phosphate - d15	TDCPP-d15	5	10	50
Tris (2-butoxyethyl) phosphate - $^{13}C_2$	M6TBEP	5	10	50
Triphenyl phosphate - $^{13}C_{18}$	MTPhP	5	10	50

Table S3. Names, acronym, molecular weight (MW) and Log K_{OW} of the targeted compounds.

**Analyte**	**Acronym**	**MW (g mol^{-1})**	**Log K_{OW}**
Tris (2-chloroethyl) phosphate	TCEP	285.5	1.6
Tris (2-choloroisopropyl) phosphate	TCPP-1.2.3	327.6	2.9
Tris (1.3-dichlor-2-propyl) phosphate	TDCIPP	430.9	3.7
Triethyl phosphate	TEP	182.2	0.9
Tripropyl phosphate	TPrP	224.2	2.1
Tris-iso-butyl phosphate	TiBP	266.3	3.6
Tri-n-butyl phosphate	TNBP	266.3	3.8
Tris (2-ehylhexyl) phosphate	TEHP	434.6	9.5
Tris (2-butoxyethyl) phosphate	TBEP	398.5	3.0
Triphenyl phosphate	TPhP	326.3	4.7
2-Ethylhexyldiphenyl phosphate	EHDPP	362.4	6.3
Tri-p-cresyl phosphate	TpCP	368.4	6.3
Tri-o-cresyl phosphate	ToCP	368.4	6.3
Tri-m-cresyl phosphate	TmCP	368.4	6.3
Tris (3.5-dimethylphenyl) phosphate	TDMPP	410.4	8.0
Tris (2-isopropylphenyl) phosphate	TPPP	452.5	9.1
Tris (4-tert-butylphenyl) phosphate	TTBPP	494.6	10.4
Tris (2.3-dibromopropyl) phosphate	TDBPP	697.6	9.1
Chlorpyrifos	Chlor	350.6	4.9
Triphenyl phosphine oxide	TPPO	278.3	**2.9**
Dioctylphenyl phosphonate	DOPP	382.5	7.5
Tributyl phosphine oxide	TBPO	218.3	3.9

Table S4. Individual OPE contribution (%) to the total pool of $\sum_{24}$OPE in the Atlantic and Southern Ocean samples.

Compound	∑TCPP	TCEP	TPhP	TEP	EHDPP	TBEP	TiBP	TNBP	TDCIPP	∑TcPs	TBPO	TPrP	TEHP	TDBPP	TPPP	Chlor	TTBPP	DOPP	TDMPP	TPPO
**Atlantic**	59.40	13.0	7.650	3.43	6.76	1.48	3.90	2.16	0.950	0.54	0.22	0.06	0.15	0.06	0.09	0.03	0.002	0.03	0.06	0.003
**Southern**	50.0	9.05	5.56	2.36	10.1	9.61	2.88	5.54	2.84	0.81	0.00	1.03	0.19	0.05	0.00	0.00	0.03	0.01	0.01	0.00
**All**	58.26	12.53	7.38	3.29	7.16	2.48	3.76	2.57	1.18	0.57	0.22	0.18	0.15	0.05	0.08	0.02	0.01	0.03	0.05	0.001

Grey area shows OPEs that on average contribute below 0.5% to the total pool and are grouped in this work as "minor-OPEs".

Table S5. Field and procedural blanks OPE abundance (ng).

Name	TEP	TPrP	TiBP	TNBP	TBPO	TCEP	TCPP1	TCPP2	TCPP3	Chlor	TDCIPP	TPhP	TBEP	EHDPP	TEHP	TPPO	ToCP	DOPP	TmCP	TpCP	TPPP	TDMPP	TDBPP	TTBPP
**ANIWOPE_010**	nd	0.01	nd	0.01	0.29	nd	0.27	0.04	nd	nd	0.01	0.03	0.11	0.02	nd	nd	nd	nd	nd	nd	nd	nd	nd	nd
**ANIWOPE_024**	nd	0.01	nd	0.02	0.05	nd	0.32	0.08	0.04	nd	0.02	0.11	nd	0.19	nd	nd	nd	nd	nd	nd	0.05	nd	0.02	nd
**ANIWOPE_050**	nd	0.03	0.07	0.06	0.11	0.16	1.32	0.30	0.02	nd	0.02	0.27	nd	0.40	nd	nd	nd	nd	nd	nd	nd	nd	nd	nd
**ANIWOPE_051**	nd	0.02	0.07	0.09	0.18	0.38	1.15	0.50	0.30	nd	0.01	0.16	nd	0.27	nd	nd	nd	nd	nd	nd	nd	nd	nd	nd
**ANIWOPE_063**	nd	0.01	nd	0.03	0.64	1.23	0.57	0.06	nd	nd	0.02	0.14	nd	0.10	nd	nd	nd	nd	nd	nd	nd	0.03	nd	nd
**ANIWOPE_064**	nd	0.01	nd	0.04	0.24	1.59	0.76	0.09	0.01	nd	0.01	0.16	nd	0.12	nd	nd	nd	nd	nd	nd	nd	nd	0.04	nd
**ANIWOPE_066**	nd	0.01	nd	0.08	0.55	nd	0.82	0.10	0.01	nd	0.06	0.33	nd	0.38	nd	nd	nd	nd	nd	nd	nd	nd	0.05	nd
**ANIWOPE_082**	nd	0.01	nd	0.04	0.40	0.28	0.45	0.11	0.01	nd	0.03	0.16	nd	0.21	nd	nd	nd	nd	nd	nd	nd	nd	nd	nd
**ANIWOPE_083**	0.01	0.02	nd	0.08	0.86	0.51	0.71	0.18	0.01	nd	nd	0.15	nd	0.15	nd	nd	nd	nd	nd	nd	nd	nd	nd	nd
**ANIWOPE_090**	nd	0.03	nd	0.03	0.14	0.30	0.91	0.51	0.03	nd	nd	0.14	nd	0.22	nd	nd	nd	nd	nd	nd	nd	nd	0.05	nd
**ANIWOPE_121**	nd	0.01	nd	0.03	0.23	5.13	0.54	0.06	nd	nd	0.02	0.07	nd	0.09	nd	nd	nd	nd	0.01	nd	0.01	0.03	nd	nd
**ANIWOPE_134**	nd	nd	nd	0.01	0.11	0.11	0.18	0.03	nd	nd	nd	0.06	nd	0.04	nd	nd	nd	nd	nd	nd	nd	0.03	0.03	nd
**ANIWOPE_140**	nd	nd	0.06	0.08	0.17	0.07	1.17	0.16	0.10	nd	nd	0.11	nd	0.10	nd	nd	nd	nd	nd	nd	nd	nd	nd	nd
**ANIWOPE_142**	nd	0.01	nd	0.02	0.12	nd	0.33	0.18	0.03	nd	nd	0.07	nd	0.03	nd	nd	nd	nd	nd	nd	nd	nd	nd	nd
**ANIWOPE_143**	nd	0.01	nd	0.25	nd	2.36	nd	2.09	0.18	nd	0.26	0.64	0.28	0.83	nd	nd	nd	nd	0.02	0.01	nd	nd	nd	nd
**ANIWOPE_174**	nd	0.01	0.02	0.04	0.23	0.02	0.40	0.04	0.02	nd	0.08	0.10	0.09	0.05	nd	nd	0.03	nd	0.02	0.01	0.01	0.04	nd	nd
**ANIWOPE_175**	nd	nd	nd	0.02	0.19	nd	0.17	0.11	0.01	nd	0.06	0.10	nd	0.09	nd	nd	nd	nd	nd	nd	nd	0.03	nd	nd
**ANIWOPE_217**	0.01	nd	0.01	0.01	0.08	nd	0.09	0.04	0.01	nd	nd	0.04	nd	0.01	nd	nd	nd	nd	nd	nd	nd	nd	nd	nd
**ANIWOPE_219**	0.01	0.01	nd	0.03	0.19	0.43	0.40	0.24	0.04	nd	nd	0.10	nd	0.03	nd	nd	nd	nd	nd	nd	nd	nd	nd	nd
**ANIWOPE_248**	0.07	0.01	0.01	0.01	nd	0.57	0.24	0.06	nd	nd	0.04	1.04	nd	0.59	nd	nd	nd	nd	nd	nd	0.09	nd	nd	nd
**ANIWOPE_PB001**	0.09	nd	0.02	0.37	nd	nd	0.10	0.02	0.13	nd	0.01	0.03	0.03	nd	nd	nd	nd	nd	nd	nd	nd	nd	nd	nd
**ANIWOPE_PB002**	0.05	nd	0.02	0.31	nd	nd	0.10	0.01	0.12	nd	0.02	0.03	0.05	0.01	nd	nd	nd	nd	nd	nd	nd	nd	nd	nd
**ANIWOPE_PB003**	nd	nd	0.03	0.36	nd	nd	0.08	0.01	0.10	nd	0.06	0.03	0.07	0.01	nd	nd	nd	nd	nd	nd	nd	nd	nd	nd
**ANIWOPE_PB004**	0.01	nd	0.04	0.45	nd	nd	0.16	0.03	0.19	nd	0.03	0.08	0.10	nd	nd	nd	nd	nd	0.01	nd	nd	nd	nd	nd
**ANIWOPE_PB005**	0.01	nd	0.03	0.51	nd	nd	0.13	nd	0.16	nd	nd	0.03	nd	nd	nd	nd	nd	nd	nd	nd	nd	nd	nd	nd
**ANIWOPE_PB006**	nd	nd	0.02	0.29	nd	nd	0.08	0.01	0.10	nd	0.01	0.03	0.05	0.01	nd	nd	nd	nd	nd	nd	nd	nd	nd	nd
**ANIWOPE_PB007**	nd	nd	0.08	0.86	nd	nd	nd	nd	0.05	nd	nd	0.02	nd	nd	nd	nd	nd	nd	nd	nd	nd	nd	nd	nd
**ANIWOPE_PB008**	nd	nd	0.02	0.24	nd	nd	0.05	0.06	0.06	nd	0.01	0.04	0.32	0.01	nd	nd	nd	nd	nd	nd	nd	nd	nd	nd
**ANIWOPE_PB009**	nd	nd	0.03	0.24	nd	nd	0.04	0.05	0.05	nd	0.01	0.04	nd	0.01	nd	nd	nd	nd	nd	nd	nd	nd	nd	nd
**ANIWOPE_PB010**	nd	nd	0.05	0.02	nd	nd	0.09	0.06	0.13	nd	0.01	0.01	nd	nd	nd	nd	nd	nd	nd	nd	nd	nd	nd	nd
**ANIWOPE_PB011**	nd	nd	0.03	0.01	nd	nd	0.05	0.02	0.07	nd	0.01	0.02	0.04	0.01	nd	nd	nd	nd	nd	nd	nd	nd	nd	nd
**ANIWOPE_PB012**	nd	nd	0.05	0.02	nd	nd	0.06	0.03	0.09	nd	0.01	0.01	0.16	nd	nd	nd	nd	nd	nd	nd	nd	nd	nd	nd
**ANIWOPE_PB013**	nd	nd	0.05	0.02	nd	nd	0.06	0.03	0.08	nd	0.01	0.01	0.07	0.01	nd	nd	nd	nd	nd	nd	nd	nd	nd	nd
**ANIWOPE_PB014**	nd	nd	0.06	0.38	nd	nd	0.07	0.09	0.09	nd	nd	0.02	0.24	nd	nd	nd	nd	nd	nd	nd	nd	nd	nd	nd
**ANIWOPE_PB015**	nd	nd	0.06	0.43	nd	nd	0.11	0.14	0.15	nd	nd	nd	nd	nd	nd	nd	nd	nd	nd	nd	nd	nd	nd	nd
**ANIWOPE_PB016**	nd	nd	0.06	0.10	nd	nd	0.03	0.03	0.04	nd	nd	nd	nd	nd	nd	nd	nd	nd	nd	nd	nd	nd	nd	nd
**ANIWOPE_PB017**	nd	nd	0.10	0.44	nd	nd	0.10	0.08	0.02	nd	nd	0.01	nd	nd	nd	nd	nd	nd	nd	nd	nd	nd	nd	nd
**ANIWOPE_PB018**	nd	nd	0.01	0.11	nd	nd	0.01	0.01	0.01	nd	0.01	0.02	nd	nd	nd	nd	nd	nd	nd	nd	nd	nd	nd	nd
**ANIWOPE_PB019**	nd	nd	0.01	0.12	nd	nd	0.01	0.01	0.01	nd	nd	0.01	nd	nd	nd	nd	nd	nd	0.06	nd	nd	nd	nd	nd
**AN2WOPE_003**	0.31	nd	0.13	nd	nd	0.04	1.35	0.12	0.06	nd	0.02	0.13	nd	nd	0.01	1.84	nd	nd	nd	nd	nd	nd	nd	nd
**AN2WOPE_015**	0.15	nd	0.06	0.05	nd	0.04	1.55	0.12	nd	nd	0.04	0.19	nd	nd	0.02	1.16	nd	nd	nd	nd	nd	nd	nd	nd
**AN2WOPE_031**	0.12	0.02	0.07	0.07	nd	0.02	0.82	0.08	0.05	nd	0.02	0.08	0.10	nd	0.01	1.17	nd	nd	nd	nd	nd	nd	nd	nd
**AN2WOPE_032**	0.06	0.02	0.04	0.04	nd	0.02	0.91	0.09	0.02	nd	0.02	0.15	0.11	nd	0.01	0.61	nd	nd	nd	nd	nd	nd	nd	nd
**AN2WOPE_033**	0.07	0.02	0.05	nd	nd	0.02	0.59	0.06	0.02	nd	nd	0.07	nd	nd	nd	1.34	nd	nd	nd	nd	nd	nd	nd	nd
**AN2WOPE_042**	0.04	0.01	0.02	0.06	nd	0.02	0.73	0.08	0.05	nd	0.01	0.09	nd	nd	nd	0.72	nd	nd	nd	nd	nd	nd	nd	nd
**AN2WOPE_048**	0.02	0.01	0.01	0.01	nd	0.02	0.19	0.03	0.03	nd	nd	0.06	0.04	nd	nd	0.18	nd	nd	nd	nd	nd	nd	nd	nd
**AN2WOPE_097**	0.07	nd	0.04	nd	nd	0.02	0.67	0.06	0.04	nd	nd	0.07	nd	nd	nd	0.86	nd	nd	nd	nd	nd	nd	nd	nd
**AN2WOPE_141**	0.21	0.01	0.02	0.01	nd	0.01	0.98	0.06	0.01	nd	0.01	0.35	nd	nd	0.01	0.28	nd	nd	nd	nd	nd	nd	nd	nd

**PB for Procedural blank, nd = non detected*

Table S6. Limits of quantification (LOQs) in ng.

OPE compound	LOQs Atlantic	LOQs Southern
**TEP**	0.064	0.272
**TPrP**	0.030	0.020
**TiBP**	0.110	0.126
**TNBP**	0.741	0.842
**TBPO**	0.713	0.001
**TCEP**	3.117	0.043
**TCPP1**	1.390	1.645
**TCPP2**	1.167	0.167
**TCPP3**	0.266	0.221
**Chlorpyrifos**	0.001	0.001
**TDCIPP**	0.153	0.052
**TPhP**	0.689	0.282
**TBEP**	0.282	0.286
**EHDPP**	0.638	0.013
**TEHP**	0.001	0.013
**TPPO**	0.001	1.846
**ToCP**	0.015	0.001
**DOPP**	0.001	0.001
**TmCP**	0.035	0.037
**TpCP**	0.007	0.001
**TPPP**	0.053	0.001
**TDMPP**	0.037	0.001
**TDBPP**	0.046	0.001
**TTBPP**	0.001	0.001

All samples consisted of 2 L of seawater, except the rain water samples that extracted 1 L of water. The limits of quantification (LOQs) were defined as the mean concentration plus three times the standard deviation of the blank response. For the analytes which were not found in procedural or field blanks, LOQs were derived from the lowest standard in the calibration curve.

Table S7. Mean surrogate (top table) and matrix spike (bottom table) recoveries (%) from the Atlantic Ocean and Southern Ocean samples.

**Mean surrogate Recoveries (%)**					
**Atlantic Ocean**					
	**D27-TNBP**	**D12-TCEP**	**D18-TCPP**	**D15-TPHP**	**D51-TEHP**
**MEAN**	45.9	68.2	28.2	88.0	127.7
**SD**	23.4	75.0	24.3	44.3	55.1
**Southern Ocean**					
	**D27-TNBP**	**D12-TCEP**	**D18-TCPP**	**D15-TPHP**	**D51-TEHP**
**MEAN**	64.9	56.7	60.7	82.2	107.0
**SD**	14.9	9.9	10.0	13.8	51.7

**Mean Matrix Spike Recoveries (%)**												
**Atlantic Ocean (N=6)**												
**Name**	**TEP**	**TiBP**	**TNBP**	**TCEP**	**TCPP**	**TDCIPP**	**TPhP**	**TBEP**	**EHDPP**	**ToCP**	**TmCP**	**TpCP**
**Mean**	18.28	43.41	31.99	55.02	21.93	67.52	64.32	95.01	144.63	81.47	94.54	138.63
**Sd**	2.79	8.20	5.07	10.51	3.68	7.12	5.48	11.29	13.25	3.39	19.89	18.30

**Southern Ocean (N=4)**												
	**TEP**	**TiBP**	**TNBP**	**TCEP**	**TCPP**	**TDCIPP**	**TPhP**	**TBEP**	**EHDPP**	**ToCP**	**TmCP**	**TpCP**
**Mean**	66.95	108.18	84.63	60.55	66.97	113.83	85.24	74.60	43.19	67.24	44.89	41.40
**Sd**	10.12	15.11	9.05	3.18	3.37	8.75	4.28	4.25	3.63	5.57	2.43	1.21

Table S8. Individual and $\sum_{24}$OPE concentration in surface and DCM samples from the Atlantic Ocean (ng L^{-1}).

station	type	depth (m)	date	lat	long	TEP	TCEP	∑TCPP	TBEP	TiBP	TDCIPP	TNBP	TPhP	EHDPP	∑TcPs	Minor-OPE	$\sum_{24}$OPES
**AN1_ST01**	5 m	4	20201218	37.92	-15.97	2.65	2.90	8.72	<loq	0.86	0.37	<loq	2.78	<loq	nd	nd	18.29
**AN1_ST01**	DCM	61	20221218	37.92	-15.97	<loq	3.81	12.38	0.18	0.23	0.35	<loq	1.88	0.46	<loq	nd	19.29
**AN1_ST02**	5 m	5	20201219	35.74	-19.07	nd	2.67	6.37	nd	0.34	nd	0.58	1.12	<loq	nd	nd	11.08
**AN1_ST02**	DCM	105	20201219	35.74	-19.07	nd	3.79	6.20	0.59	2.41	0.85	0.97	5.62	1.67	0.31	nd	22.41
**AN1_ST03**	5 m	5	20201220	35.74	-19.07	3.32	<loq	5.55	0.21	0.27	0.23	<loq	3.04	1.23	0.06	nd	13.91
**AN1_ST03**	DCM	115	20201220	35.74	-19.07	1.44	1.63	6.24	0.21	0.14	0.19	<loq	1.45	0.41	nd	nd	11.70
**AN1_ST04**	5 m	5	20201221	28.66	-28.41	<loq	2.00	5.99	0.20	0.51	0.59	<loq	1.18	<loq	nd	nd	10.48
**AN1_ST04**	DCM	86	20201221	28.66	-28.41	<loq	2.21	7.91	0.33	0.47	0.62	<loq	1.87	0.49	nd	nd	13.88
**AN1_ST05**	5 m	5	20201222	25.63	-24.90	0.88	1.57	5.31	0.22	0.13	0.24	<loq	1.70	0.35	<loq	nd	10.39
**AN1_ST05**	DCM	120	20201222	25.63	-24.90	<loq	<loq	3.74	0.23	0.14	0.17	<loq	1.35	<loq	0.03	nd	5.67
**AN1_ST06**	5 m	5	20201223	22.22	-26.63	<loq	3.06	5.78	0.22	0.18	0.25	1.20	4.28	0.64	0.43	0.03	16.08
**AN1_ST07**	5 m	5	20201224	18.87	-28.32	<loq	4.00	13.61	0.22	0.43	0.41	<loq	2.30	<loq	nd	nd	20.98
**AN1_ST07**	DCM	85	20201224	18.87	-28.32	<loq	3.88	29.10	0.25	0.42	0.16	<loq	1.22	0.54	nd	nd	35.58
**AN1_ST08**	5 m	5	20201225	14.97	-28.70	0.70	5.90	7.78	<loq	0.20	0.08	<loq	1.62	<loq	nd	nd	16.28
**AN1_ST09**	5 m	5	20201226	10.87	-29.23	1.21	7.42	9.10	0.17	0.61	0.26	1.08	3.15	0.71	0.40	nd	24.09
**AN1_ST09**	DCM	50	20201226	10.87	-29.23	0.92	11.01	12.68	0.20	0.24	0.41	<loq	3.15	0.84	0.16	nd	29.62
**AN1_ST10**	5 m	5	20201227	7.35	-29.58	<loq	1.61	11.29	<loq	0.44	0.11	<loq	1.16	0.40	<loq	nd	15.01
**AN1_ST11**	5 m	5	20201228	3.83	-29.86	0.07	5.12	5.68	<loq	0.38	0.19	<loq	1.41	0.41	nd	nd	13.27
**AN1_ST11**	DCM	77	20201228	3.83	-29.86	<loq	2.92	4.49	<loq	0.10	0.21	<loq	1.11	<loq	nd	nd	8.83
**AN1_ST12**	5 m	5	20201229	0.14	-30.50	8.20	3.37	25.20	<loq	2.97	0.49	<loq	3.13	0.63	nd	nd	43.98
**AN1_ST12**	DCM	48	20201229	0.14	-30.50	<loq	2.05	13.77	<loq	0.79	<loq	<loq	1.97	0.36	<loq	nd	18.94
**AN1_ST13**	5 m	5	20201230	-2.98	-30.95	0.47	2.65	17.74	0.44	0.39	0.42	<loq	4.81	0.94	0.81	nd	28.72
**AN1_ST14**	5 m	5	20201231	-6.78	-31.33	0.32	<loq	4.39	<loq	0.33	0.33	<loq	1.76	0.67	nd	nd	7.82
**AN1_ST14**	DCM	120	20201231	-6.78	-31.33	0.06	<loq	3.97	<loq	0.35	0.22	<loq	1.26	0.33	nd	nd	6.19
**AN1_ST15**	5 m	5	20210102	-14.37	-33.92	nd	3.87	70.56	0.21	0.47	0.22	<loq	2.64	0.53	nd	nd	78.50
**AN1_ST15**	DCM	110	20210102	-14.37	-33.92	<loq	<loq	8.41	<loq	0.23	0.14	<loq	1.66	0.47	<loq	nd	10.91
**AN1_ST16**	5 m	5	20210103	-17.78	-35.27	nd	8.19	27.87	2.07	0.72	nd	4.85	2.57	0.51	nd	nd	46.77
**AN1_ST17**	5 m	5	20210104	-21.30	-36.80	0.04	5.36	7.64	0.65	0.25	0.15	<loq	2.75	1.90	0.87	0.22	19.83
**AN1_ST17**	DCM	90	20210104	-21.30	-36.80	<loq	4.40	13.62	0.36	0.14	0.12	<loq	1.82	0.51	0.06	0.04	21.07
**AN1_ST18**	5 m	5	20210105	-24.91	-38.72	0.53	2.84	18.37	2.80	0.30	0.11	2.27	5.05	6.06	4.21	0.01	42.56
**AN1_ST18**	DCM	55	20210105	-24.91	-38.72	7.80	nd	11.56	nd	0.14	<loq	<loq	1.83	0.41	0.15	nd	21.90
**AN1_ST19**	5 m	5	20210106	-27.79	-40.65	0.50	nd	8.94	nd	0.95	nd	2.70	nd	0.61	nd	nd	13.71
**AN1_ST20**	5 m	5	20210107	-30.98	-42.96	2.40	2.10	13.35	0.54	0.11	0.33	<loq	2.30	<loq	nd	nd	21.12
**AN1_ST20**	DCM	110	20210107	-30.98	-42.96	<loq	<loq	10.57	<loq	0.08	0.10	<loq	2.31	<loq	<loq	nd	13.05
**AN1_ST21**	5 m	5	20210108	-34.40	-45.63	<loq	<loq	1.74	<loq	<loq	nd	<loq	0.38	<loq	nd	nd	2.12
**AN1_ST22**	5 m	5	20210109	-37.24	-48.09	<loq	<loq	8.51	<loq	0.20	0.09	<loq	nd	<loq	0.07	nd	8.87
**AN1_ST22**	DCM	60	20210109	-37.24	-48.09	0.33	<loq	2.37	<loq	0.23	<loq	<loq	0.75	<loq	nd	nd	3.68
**AN1_ST23**	5 m	5	20210111	-43.79	-54.75	nd	<loq	18.48	nd	0.63	nd	<loq	nd	nd	nd	nd	19.11
**AN1_ST24**	5 m	5	20210112	-46.79	-58.46	11.35	8.63	7.74	nd	0.24	0.51	<loq	3.00	<loq	0.39	nd	31.87
**AN1_ST24**	DCM	34	20210112	-46.79	-58.46	3.92	nd	5.51	0.19	0.06	nd	<loq	0.77	<loq	nd	nd	10.44

*Category "Minor-OPE" results from the sum of: TEHP, TPrP, TPPP, TBPO, Chlorpyrifos, DOPP, TPPO, TDMPP, TDBPP and TTBPP; nd = non detected; <loq = value below limit of quantification; ∑TCPP = sum of three TCPP isomers (TCPP1, TCPP2 and TCPP3); ∑TcPs = sum of three cresyl isomers (TpC, TmCP and TpCP).

Table S9. Mean and SD of 5 m depth and DCM concentrations (ng L^{-1}) in the three ocean basins sampled.

	North Atlantic				South Atlantic				Southern Ocean			
	5 m		DCM		5 m		DCM		5 m		DCM	
	Mean	SD	Mean	SD	Mean	SD	Mean	SD	Mean	SD	Mean	SD
**TEP**	1.43	2.40	0.29	0.52	1.31	3.23	1.74	3.03	0.21	0.11	0.25	0.11
**TiBP**	0.61	0.77	0.55	0.73	0.39	0.27	0.18	0.10	0.24	0.13	0.25	0.11
**TNBP**	0.52	0.30	0.44	0.20	1.10	1.44	0.37	0.00	0.52	0.36	0.49	0.15
**TCEP**	3.43	1.88	3.65	2.59	3.45	2.59	1.97	1.07	0.60	0.29	0.74	0.35
**∑TCPP**	9.20	5.66	10.70	7.82	17.10	18.40	8.00	4.19	5.10	2.94	3.65	1.54
**TDCIPP**	0.28	0.16	0.34	0.25	0.21	0.15	0.12	0.05	0.21	0.14	0.34	0.57
**TPhP**	2.24	1.03	2.18	1.43	2.19	1.65	1.49	0.58	0.54	0.33	0.42	0.26
**TBEP**	0.17	0.04	0.25	0.14	0.63	0.88	0.18	0.08	0.72	0.88	0.86	0.71
**EHDPP**	0.50	0.27	0.60	0.43	1.07	1.64	0.38	0.08	0.76	0.60	0.65	0.50
**∑TcPs**	0.27	0.18	0.27	0.19	0.14	0.06	0.12	0.02	0.09	0.07	0.14	0.14
**Minor**	0.09	0.15	0.07	0.10	0.54	1.20	0.04	0.05	0.14	0.32	0.01	0.01
**$\sum_{24}$OPE**	18.60	9.07	19.30	9.29	27.60	20.30	14.50	6.47	9.00	4.53	7.77	2.79

Table S10. Individual and $\sum_{24}$OPE concentration in surface and DCM samples from the Southern Ocean (ng L^{-1}).

Station	Type	Depth(m)	Date	LAT	LONG	TEP	TCEP	∑TCPP	TBEP	TiBP	TDCIPP	TNBP	TPHP	EHDPP	∑TcPs	Minor-OPE	$\sum_{24}$OPE
**AN2_ST01**	5 m	5.44	220123	-62.71	-60.64	0.30	0.72	4.01	0.22	0.33	0.13	1.73	0.38	0.14	0.01	0.09	8.06
**AN2_ST01**	DCM	25.19	220123	-62.71	-60.64	0.23	0.70	3.00	0.32	0.39	0.16	0.88	0.46	0.08	0.01	0.15	6.36
**AN2_ST02**	5 m	4.81	220124	-62.92	-61.04	0.25	0.80	6.91	0.64	0.46	0.18	<loq	1.24	0.44	0.00	0.18	11.10
**AN2_ST02**	DCM	29.48	220124	-62.92	-61.04	0.45	1.33	4.83	0.64	0.41	0.21	0.57	1.04	0.67	0.04	0.09	10.28
**AN2_ST03**	5 m	4.81	220125	-64.58	-62.57	<loq	0.53	3.23	0.24	0.13	0.51	<loq	0.48	0.24	0.00	0.01	5.37
**AN2_ST04**	5 m	4.55	220126	-65.15	-65.64	<loq	0.43	2.97	0.25	0.17	0.11	<loq	0.27	0.16	0.01	0.05	4.41
**AN2_ST04**	DCM	19.60	220126	-65.15	-65.64	<loq	0.69	2.82	0.26	0.25	0.10	<loq	0.24	0.21	0.00	0.05	4.62
**AN2_ST05**	5 m	7.44	220127	-66.92	-69.10	0.41	0.93	6.29	1.21	0.23	0.22	<loq	0.81	1.36	0.01	0.03	11.50
**AN2_ST05**	DCM	19.84	220127	-66.92	-69.10	0.36	0.43	3.24	1.70	0.13	0.11	<loq	0.34	1.14	0.00	0.05	7.51
**AN2_ST06**	5 m	5.06	220128	-67.75	-68.03	0.36	0.34	3.15	0.64	0.25	0.29	<loq	0.30	1.52	0.01	0.12	6.97
**AN2_ST07**	5 m	4.63	220130	-70.85	-79.89	0.39	0.33	4.00	0.29	0.20	0.09	<loq	0.39	0.40	0.00	0.26	6.35
**AN2_ST07**	DCM	21.90	220130	-70.85	-79.89	0.30	0.44	3.48	0.46	0.15	1.84	<loq	0.36	0.31	0.01	0.41	7.76
**AN2_ST08**	5 m	4.65	220130	-70.85	-79.89	0.30	0.57	4.73	0.67	0.42	0.25	<loq	0.51	1.72	0.56	0.11	9.83
**AN2_ST09**	5 m	5.45	220131	-69.21	-78.84	<loq	0.88	8.06	0.48	0.31	0.45	<loq	0.55	1.61	0.06	0.11	12.52
**AN2_ST09**	DCM	30.40	220131	-69.21	-78.84	<loq	0.84	6.14	0.57	0.32	0.28	<loq	0.48	1.02	0.03	0.34	10.03
**AN2_ST10**	5 m	5.77	220201	-67.70	-75.00	<loq	0.69	3.96	0.79	0.11	0.19	<loq	0.38	0.70	0.01	0.05	6.87
**AN2_ST10**	DCM	49.44	220201	-67.70	-75.00	0.28	1.11	5.03	2.03	0.25	0.16	<loq	0.40	0.76	0.01	0.05	10.08
**AN2_ST11**	5 m	4.94	220204	-62.57	-58.62	nd	1.16	12.57	3.46	0.36	0.24	<loq	1.20	1.01	0.02	0.08	20.09
**AN2_ST11**	DCM	37.43	220204	-62.57	-58.62	<loq	0.90	3.49	1.59	0.23	0.17	<loq	0.32	1.51	0.01	0.02	8.25
**AN2_ST13**	5 m	4.99	220205	-62.97	-60.29	<loq	0.23	<loq	0.27	<loq	0.07	<loq	0.30	0.45	0.09	0.01	1.42
**AN2_ST13**	DCM	57.83	220205	-62.97	-60.29	0.18	0.22	<loq	<loq	0.08	0.03	<loq	<loq	0.13	0.00	0.02	0.67
**AN2_ST14**	5 m	5.00	220206	-62.95	-60.64	<loq	0.22	5.56	nd	0.09	0.03	<loq	0.27	0.14	1.07	0.04	7.42

* Category "Minor-OPEs" results from the sum of: TEHP, TPrP, TPPP, TBPO, Chlorpyrifos, DOPP, TDMPP, TPPO, TDBPP and TTBPP; nd = non detected; <loq = value below limit of quantification; ∑TCPP = sum of three TCPP isomers (TCPP1, TCPP2 and TCPP3); ∑TcPs = sum of three cresyl isomers (TpC, TmCP and TpCP).

Table S11. Environmental variables measured at surface waters and atmospheric conditions in the Atlantic and Southern Oceans.

Station	Depth	Ocean	Latitude	Longitude	Wind speed	Water T°	Air T°	Humidity	Solar Radiation	Atmospheric Pressure	Bacterial Abundance	Bacterial Production	Chl *a*	Salinity
Units					$m\ s^{-1}$	°C	°C	%	$W\ m^{-2}$	hPa	cells mL^{-1}	pmol Leu $L^{-1}\ h^{-1}$	μg L^{-1}	PSU
**AN1_ST01**	5 m	Atlantic	37.92	-15.97	11.0	17.5	17.2	90.0	17.1	1027.9	6.47E+05	4.5	0.2	36.3
**AN1_ST02**	5 m	Atlantic	35.74	-19.07	4.3	17.8	17.2	72.1	100.6	1031.6	5.81E+05	8.2	0.1	36.3
**AN1_ST03**	5 m	Atlantic	32.18	-19.07	0.7	19.9	19.4	78.8	48.7	1034.5	5.27E+05	8.4	0.1	36.8
**AN1_ST04**	5 m	Atlantic	28.66	-28.41	4.4	21.4	21.0	66.2	368.3	1034.2	5.00E+05	8.7	0.1	37.1
**AN1_ST05**	5 m	Atlantic	25.63	-24.90	6.4	22.9	21.0	71.8	23.7	1030.9	6.39E+05	2.8	0.1	37.3
**AN1_ST06**	5 m	Atlantic	22.22	-26.63	3.0	23.6	21.8	56.6	38.9	1027.2	5.57E+05	12.5	0.2	37.0
**AN1_ST07**	5 m	Atlantic	18.87	-28.32	6.1	24.0	22.3	67.3	42.6	1023.2	6.45E+05	7.3	0.2	36.9
**AN1_ST08**	5 m	Atlantic	14.97	-28.70	4.0	25.5	23.2	65.3	132.2	1022.5	7.99E+05	14.7	0.2	35.5
**AN1_ST09**	5 m	Atlantic	10.87	-29.23	8.8	26.4	25.1	77.7	4.5	1020.6	7.85E+05	16.8	0.2	35.6
**AN1_ST10**	5 m	Atlantic	7.35	-29.58	2.0	27.5	26.5	82.2	185.9	1021.6	9.72E+05	23.9	0.2	35.0
**AN1_ST11**	5 m	Atlantic	3.83	-29.86	1.4	27.9	25.5	87.6	20.8	1021.1	8.18E+05	18.4	0.2	35.4
**AN1_ST12**	5 m	Atlantic	0.14	-30.50	7.7	27.0	26.7	80.2	120.8	1020.8	7.66E+05	5.7	0.2	36.3
**AN1_ST13**	5 m	Atlantic	-2.98	-30.95	9.2	26.9	26.6	81.4	135.3	1020.3	8.31E+05	16.3	0.1	36.2
**AN1_ST14**	5 m	Atlantic	-6.78	-31.33	9.1	27.5	27.0	74.3	295.7	1022.2	7.29E+05	13.7	0.1	36.5
**AN1_ST15**	5 m	Atlantic	-14.37	-33.92	5.2	26.9	26.7	64.2	241.2	1023.9	3.53E+05	21.5	0.1	37.3
**AN1_ST16**	5 m	Atlantic	-17.78	-35.27	7.3	27.5	26.6	69.2	514.5	1023.4	1.47E+06	32.2	0.5	37.3
**AN1_ST17**	5 m	Atlantic	-21.30	-36.80	7.7	27.2	26.5	77.1	434.2	1024.8	1.45E+06	16.2	0.2	37.2
**AN1_ST18**	5 m	Atlantic	-24.91	-38.72	11.6	26.2	26.5	77.3	221.1	1023.9	7.41E+05	18.3	0.2	36.3
**AN1_ST19**	5 m	Atlantic	-27.79	-40.65	3.2	24.4	24.8	89.3	160.2	1023.2	7.42E+05	26.8	0.1	36.3
**AN1_ST20**	5 m	Atlantic	-30.98	-42.96	2.6	23.6	21.7	86.9	44.9	1020.6	7.08E+05	17.4	0.1	36.1
**AN1_ST21**	5 m	Atlantic	-34.40	-45.63	6.5	23.9	23.6	80.2	358.9	1018.6	7.55E+05	14.9	0.1	37.0
**AN1_ST22**	5 m	Atlantic	-37.24	-48.09	9.6	19.4	19.0	65.8	322.6	1021.0		27.5	0.2	35.1
**AN1_ST23**	5 m	Atlantic	-43.79	-54.75	5.0	18.5	17.7	81.0	157.2	1014.6	2.29E+06	98.6	0.2	34.2
**AN1_ST24**	5 m	Atlantic	-46.79	-58.46	2.6	13.7	12.2	67.2	187.0	1015.6	2.64E+06	65.8	0.5	34.2
**AN2_ST01**	5 m	Southern	-62.71	-60.64	8.1	1.4	1.9	97.6	136.6	987.7	4.30E+05	29.2	0.7	34.1
**AN2_ST02**	5 m	Southern	-62.92	-61.04	1.8	1.4	1.3	96.7	122.5	982.4	4.12E+05	22.3	0.9	34.1
**AN2_ST03**	5 m	Southern	-64.58	-62.57	9.8	1.0	1.5	92.1	456.0	985.6	2.39E+05	5.3	1.2	34.0
**AN2_ST04**	5 m	Southern	-65.15	-65.64	6.3	1.0	0.2	81.4	217.7	990.8	2.15E+05	41.4	1.9	33.5
**AN2_ST05**	5 m	Southern	-66.92	-69.10	10.7	0.5	0.5	94.5	107.6	989.5	2.26E+05	86.2	2.9	33.4
**AN2_ST06**	5 m	Southern	-67.75	-68.03	3.6	0.3	0.0	81.5	179.3	998.9	2.13E+05	39.6	2.6	33.1
**AN2_ST07**	5 m	Southern	-70.85	-79.89	6.8	0.0	0.0	97.9	124.0	973.8	5.93E+05	57.1	3.6	33.1
**AN2_ST08**	5 m	Southern	-70.85	-79.89	6.8	0.0	0.0	92.6	182.0	975.2	5.58E+05	121.1	4.7	33.2
**AN2_ST09**	5 m	Southern	-69.21	-78.84	10.2	0.0	0.0	96.7	106.7	971.4	2.77E+05	34.0	0.7	33.6
**AN2_ST10**	5 m	Southern	-67.70	-75.00	11.4	0.7	0.0	88.2	45.6	969.5	1.77E+05	9.9	1.8	33.9
**AN2_ST11**	5 m	Southern	-62.57	-58.62	12.8	1.3	1.8	93.4	107.6	993.7	4.81E+05	48.7	2.7	34.3
**AN2_ST13**	5 m	Southern	-62.97	-60.29	7.9	0.3	1.5	98.8	106.9	996.0	2.45E+05	16.5	0.6	34.4
**AN2_ST14**	5 m	Southern	-62.95	-60.64	11.4	1.7	1.1	95.9	61.3	992.2	1.09E+06	27.2	1.0	34.0

Table S12. Summary of the polynomial regression models applied to log-transformed grouped OPE concentrations across different depths (5 m, DCM, 1% PAR, MOx, and Deep) with latitude.

Cl-OPEs	5 m	p	DCM	p	1%PAR	p	MOx	p	Deep	p
**(a) Intercept**	2.39	**< 0.001**	2.19	**< 0.001**	2.20	**< 0.001**	2.27	**< 0.001**	2.38	**< 0.001**
**(b) $(lat)^1$**	1.64	**0.003**	1.84	**< 0.001**						
**(c) $(lat)^2$**	-2.11	**< 0.001**							-1.16	**0.03**
**Residual SD**	0.5		0.48		0.86		0.57		0.43	
**Multiple R^2**	0.42		0.43		0.30		0.33		0.59	
**F-statistic**	12.8		8.54		1.93		2.03		5.67	
**P-value**	**< 0.001**		**0.002**		0.20		0.19		**0.03**	

Aryl-OPE	5 m	p	DCM	p	1%PAR	p	MOx	p	Deep	p
**(a) Intercept**	1.14	**< 0.001**	0.99	**< 0.001**	1.10	**< 0.001**	1.11	**< 0.001**	1.08	**< 0.001**
**(b) $(lat)^1$**			1.26	**< 0.001**						
**(c) $(lat)^2$**										
**Residual SD**	0.46		0.31		0.64		0.27		0.17	
**Multiple R^2**	0.21		0.42		0.21		0.08		0.07	
**F-statistic**	4.68		8.17		1.21		0.39		0.29	
**P-value**	**0.01**		**0.002**		0.34		0.69		0.76	

Alkyl-OPE	5 m	p	DCM	p	1%PAR	p	MOx	p	Deep	p
**(a) Intercept**	1.10	**< 0.001**	0.95	**< 0.001**	0.87	**< 0.001**	1.15	**0.001**	0.75	**< 0.001**
**(b) $(lat)^1$**									-0.69	**< 0.001**
**(c) $(lat)^2$**									0.41	**< 0.001**
**Residual SD**	0.54		0.44		0.32		0.68		0.12	
**Multiple R^2**	0.03		0.04		0.25		0.10		0.84	
**F-statistic**	0.68		0.42		1.51		0.44		21.5	
**P-value**	0.51		0.65		0.27		0.65		**< 0.001**	

Minor-OPE	5 m	p	DCM	p	1%PAR	p	MOx	p	Deep	p
**(a) Intercept**	0.14	**< 0.001**	0.15	**< 0.001**	0.13	**< 0.001**	0.16	**< 0.001**	0.18	**< 0.001**
**(b) $(lat)_1$**	0.41	**< 0.001**	0.29	**0.002**	0.05	**< 0.001**	0.23	**0.005**		
**(c) $(lat)_2$**	0.27	**< 0.001**	0.33	**0.001**	0.16	**0.013**	0.23	**0.006**	0.29	**0.01**
**Residual SD**	0.07		0.09		0.05		0.06		0.09	
**Multiple R^2**	0.58		0.53		0.9		0.77		0.59	
**F-statistic**	23.8		12.7		23.12		13.64		5.87	
**P-value**	**< 0.001**		**< 0.001**		**0.0002**		**0.002**		**0.02**	

The table shows the significant estimated coefficients for the intercept and the two polynomial terms of latitude, along with their corresponding p-values (p) for different depths and compounds. For each model, we fitted a quadratic polynomial regression equation of the form: $\mathbf{OPE = Intercept + a \cdot (lat)_1 + b \cdot (lat)_2}$ *where* lat_1 *and* lat_2 *are the first and second polynomial terms of latitude, respectively.*

The residual standard deviation (SD), multiple R-squared, F-statistic, and overall model p-value are also provided for each depth. To interpret the coefficients, note that significant p-values ($p < 0.05$), which are bolded in the table, indicate a meaningful relationship between latitude and Cl-OPE concentrations at the given depth.

Table S13. Wet Deposition and related surface sample concentrations (in ng L^{-1}) of individual and $\sum_{24}$OPE in the Atlantic Ocean, and calculation of EF_{WD} (C_{WD}/C_{SURF}) for those samples.

Sample code	Date	LAT	LONG	TEP	TCEP	∑TCPP	TBEP	TiBP	TDCIPP	TNBP	TPHP	EHDPP	∑TcPs	Minor	$\sum_{24}$OPE
AN1_ST10_surface	20201227	7.3	-29.6	<loq	1.61	11.29	<loq	0.44	0.11	<loq	1.16	0.40	0.01	nd	15.01
AN1_ST10_WD	20201227	7.3	-29.6	<loq	16.33	270.39	5.83	24.18	4.21	7.15	16.42	51.51	0.16	1.96	398.14
AN1_ST11_surface	20201228	3.8	-29.8	0.07	5.12	5.68	<loq	0.38	0.19	<loq	1.41	0.41	nd	nd	13.27
AN1_ST11_WD	20201228	3.8	-29.8	0.23	12.15	288.46	8.53	35.22	1.39	3.79	15.10	55.74	0.16	0.80	421.58

**nd non detected; <loq: concentration below limit of quantification.*

Table S14. Atmospheric aerosol concentrations of $\sum_{14}$OPEs (ng m^{-3}) from *Malaspina* (Castro-Jiménez et al., 2016) from latitudes ranging between 7°N and 0°N (top table) and rain water $\sum_{14}$OPEs (ng m^{-3}) concentrations from AN1_ST10 and AN1_ST11 2021 Atlantic campaign.

Atmospheric Aerosol (ng m^{-3})												
**Sample**	**LAT**	**LONG**	**TCEP**	**TDCP**	**∑TCPP**	**TiBP**	**TnBP**	**TPhP**	**EHDPP**	**TEHP**	**∑TCPs**	**$\sum_{14}$OPEs**
MA6	6.06	-25.9	0.08	0.10	0.96	0.07	0.44	0.01	0.18	0.41	0.01	2.24
MA7	0.29	-26.0	0.16	0.06	0.71	0.13	0.37	0.01	0.18	0.31	0.01	1.93

Rainwater (ng m^{-3})												
**Sample**	**LAT**	**LONG**	**TCEP**	**TDCP**	**∑TCPPs**	**TiBP**	**TnBP**	**TPhP**	**EHDPP**	**TEHP**	**∑TCPs**	**$\sum_{14}$OPEs**
**AN1_ST10**	7.35	-29.58	16326.0	4211.7	270391.0	24176.8	7150.0	16420.4	51508.0	1957.9	158.7	392300.0
**AN1_ST11**	3.83	-29.86	12154.8	1391.2	288457.0	35219.0	3790.4	15097.6	55742.2	803.0	163.3	412818.0

Table S15. *Log* K_{RP} values from the Atlantic Ocean.

**LogK$_{RP}$**										
**Site**	**TCEP**	**TDCIPP**	**∑TCPP**	**TiBP**	**TNBP**	**TPhP**	**EHDPP**	**TEHP**	**∑TcPs**	**∑$_{14}$OPEs**
**1**	5,3	4,6	5,4	5,5	4,2	6,2	5,5	3,7	4,1	5,2
**2**	4,9	4,3	5,6	5,4	4,0	6,1	5,5	3,4	4,2	5,3
**MEAN**	5,1	4,5	5,5	5,5	4,1	6,1	5,5	3,5	4,2	5,3
**SD**	0,3	0,2	0,1	0,1	0,1	0,0	0,0	0,2	0,0	0,1

Table S16. Mean ± SD concentrations for different OPEs groups (ng L^{-1}) by ocean and depth.

	Atlantic Ocean (N=7)				Southern Ocean (N=4)			
	**Cl-OPEs**	**Aryl-OPEs**	**Alkyl-OPEs**	**Minor-OPEs**	**Cl-OPEs**	**Aryl-OPEs**	**Alkyl-OPEs**	**Minor-OPEs**
**Surface**	16.8 ± 14.4	3.31 ± 3.01	3.08 ± 3.26	0.20 ± 0.15	5.91 ± 3.21	1.45 ± 0.76	1.69 ± 0.96	0.09 ± 0.07
**DCM**	12.7 ± 7.62	2.44 ± 1.55	1.94 ± 2.09	0.20 ± 0.16	4.73 ± 1.90	1.08 ± 0.60	1.84 ± 0.73	0.13 ± 0.14
**1%PAR**	17.7 ± 20.0	3.55 ± 4.05	1.47 ± 1.17	0.20 ± 0.16	5.12 ± 1.98	1.43 ± 0.74	1.68 ± 0.18	0.05 ± 0.02
**MOx**	14.2 ± 11.6	2.36 ± 0.76	3.62 ± 4.20	0.21 ± 0.17	5.63 ± 2.11	1.85 ± 1.06	1.91 ± 0.46	0.12 ± 0.08
**Deep**	16.4 ± 14.1	2.07 ± 0.47	0.75 ± 0.11	0.21 ± 0.17	5.54 ± 0.56	1.83 ± 0.56	2.00 ± 0.34	0.21 ± 0.18

Table S17. Individual and $\sum_{24}$OPE concentration in vertical profiles from the Atlantic Ocean (ng L^{-1}).

Station Code	Depth Code	Depth (m)	Date (yymmdd)	LAT	LONG	TEP	TCEP	∑TCPP	TBEP	TiBP	TDCIPP	TNBP	TPhP	EHDPP	∑TcPs	Minor-OPE	$\sum_{24}$OPE
	SURF	5	201218	37.92	-15.97	2.65	2.90	8.72	<loq	0.86	0.37	<loq	2.78	<loq	nd	nd	18.3
	DCM	61	201218	37.92	-15.97	<loq	3.81	12.38	0.18	0.23	0.35	<loq	1.88	0.46	<loq	nd	19.23
**AN1_ST01**	1%PAR	115	201218	37.92	-15.97	nd	nd	nd	nd	nd	nd	nd	nd	nd	nd	nd	0.00
	MOx	848	201218	37.92	-15.97	<loq	4.21	11.04	0.23	0.54	0.65	<loq	2.18	0.57	<loq	nd	19.4
	DEEP	2000	201218	37.92	-15.97	nd	2.38	6.74	0.21	0.21	0.17	<loq	2.31	<loq	0.03	nd	12.0
	SURF	5	201221	28.66	-28.41	<loq	2.00	5.99	0.20	0.51	0.59	<loq	1.18	<loq	nd	nd	10.5
	DCM	125	201221	28.66	-28.41	<loq	2.21	7.91	0.33	0.47	0.62	<loq	1.87	0.49	nd	nd	13.9
**AN1_ST04**	1%PAR	86	201221	28.66	-28.41	<loq	<loq	2.50	<loq	0.15	0.12	<loq	0.88	<loq	nd	nd	3.65
	MOx	900	201221	28.67	-28.41	9.99	1.79	5.88	<loq	0.10	0.16	<loq	1.42	<loq	nd	nd	19.3
	DEEP	2000	201221	28.67	-28.41	<loq	1.69	6.64	<loq	0.24	0.40	<loq	1.29	<loq	nd	nd	10.3
	SURF	5	201226	10.87	-29.23	1.21	7.42	9.10	0.17	0.61	0.26	1.08	3.15	0.71	0.40	nd	24.1
	DCM	55	201226	10.87	-29.23	0.92	11.01	12.68	0.20	0.24	0.41	<loq	3.15	0.84	0.16	nd	29.6
**AN1_ST09**	1%PAR	50	201226	10.87	-29.23	1.48	6.29	13.18	0.16	0.45	0.33	<loq	2.49	0.76	<loq	nd	25.1
	MOx	400	201226	10.04	-29.33	<loq	2.41	13.98	<loq	0.26	0.11	<loq	1.70	<loq	<loq	nd	18.4
	DEEP	2000	201226	10.04	-29.33	<loq	11.03	5.21	<loq	0.13	0.10	<loq	1.45	0.33	<loq	nd	18.2
	SURF	5	201229	0.14	-30.50	8.20	3.37	25.20	<loq	2.97	0.49	<loq	3.13	0.63	nd	nd	44.0
	DCM	48	201229	0.14	-30.50	<loq	2.05	13.77	<loq	0.79	<loq	<loq	1.97	0.36	<loq	nd	18.9
**AN1_ST12**	1%PAR	78	201229	0.14	-30.50	0.05	3.55	10.62	0.19	0.49	0.50	<loq	2.36	0.47	nd	nd	18.2
	MOx	400	201229	0.14	-30.52	8.01	1.90	4.18	<loq	0.22	0.13	<loq	2.08	<loq	<loq	nd	16.5
	DEEP	2000	201229	0.14	-30.52	<loq	3.78	6.90	<loq	0.10	0.15	<loq	1.31	0.32	<loq	nd	12.6
	SURF	5	210102	-14.37	-33.92	nd	3.87	70.56	0.21	0.47	0.22	<loq	2.64	0.53	nd	nd	78.5
	DCM	110	210102	-14.37	-33.92	<loq	<loq	8.41	<loq	0.23	0.14	<loq	1.66	0.47	<loq	nd	10.9
**AN1_ST15**	1%PAR	145	210102	-14.37	-33.92	0.07	nd	1.27	nd	<loq	nd	<loq	<loq	<loq	nd	nd	1.34
	MOx	350	210102	-14.37	-33.93	1.52	2.61	35.75	<loq	0.29	0.08	<loq	1.87	<loq	<loq	nd	42.1
	DEEP	2000	210102	-14.37	-33.93	0.06	6.12	41.54	<loq	0.35	0.29	<loq	1.93	0.32	0.05	nd	50.7
	SURF	5	210105	-24.91	-38.72	0.53	2.84	18.37	2.80	0.30	0.11	2.27	5.05	6.06	4.21	nd	42.5
	DCM	55	210105	-24.91	-38.72	7.80	nd	11.56	nd	0.14	<loq	<loq	1.83	0.41	0.15	nd	21.9
**AN1_ST18**	1%PAR	80	210105	-24.91	-38.72	<loq	28.59	29.58	0.16	0.37	1.01	0.98	10.07	0.86	1.48	nd	73.1
	MOx	570	210105	-24.94	-38.72	0.24	<loq	8.03	0.39	0.13	0.11	<loq	2.69	0.80	0.32	nd	12.7
	DEEP	2000	210105	-24.94	-38.72	<loq	<loq	8.41	0.16	0.25	0.18	<loq	2.20	0.44	0.03	nd	11.7
	SURF	5	210109	-37.24	-48.09	<loq	<loq	8.51	<loq	0.20	0.09	<loq	nd	<loq	0.07	nd	8.87
	DCM	60	210109	-37.24	-48.09	0.33	<loq	2.37	<loq	0.23	<loq	<loq	0.75	<loq	nd	nd	3.68
**AN1_ST22**	1%PAR	70	210109	-37.24	-48.09	<loq	1.61	2.99	nd	0.30	0.08	<loq	0.65	<loq	nd	nd	5.63
	MOx	375	210109	-37.24	-48.09	<loq	<loq	3.02	<loq	0.06	<loq	<loq	1.15	0.33	<loq	nd	4.57
	DEEP	2000	210109	-37.24	-48.09	0.07	4.62	6.75	<loq	0.06	0.16	<loq	1.42	0.34	<loq	nd	13.4

*Category "minor-OPE" results from the sum of: TEHP, TPrP, TPPP, TBPO, TPPO, Chlorpyrifos, DOPP, TDMPP, TDBPP and TTBPP; nd = non detected; <loq = value below limit of quantification; ∑TCPP = sum of three TCPP isomers (TCPP1, TCPP2 and TCPP3); ∑TcPs = sum of three cresyl isomers (TpCP, TmCP and TpCP).

Table S18. Individual and $\sum_{24}$OPE concentration in vertical profiles from the Southern Ocean (ng L^{-1}).

Station Code	Depth Code	Depth (m)	Date (yymmdd)	LAT	LONG	TEP	TCEP	∑TCPP	TBEP	TiBP	TDCIPP	TNBP	TPHP	EHDPP	∑TcPs	Minor-OPE	$\sum_{24}$OPE
**AN2_ST05**	SURF	7	220127	-66.92	-69.10	0.41	0.93	6.29	1.21	0.23	0.22	<loq	0.81	1.36	0.01	0.00	11.47
	DCM	19	220127	-66.92	-69.10	0.36	0.43	3.24	1.70	0.13	0.11	<loq	0.34	1.14	0.00	0.00	7.45
	1%PAR	130	220127	-66.92	-69.10	0.19	0.66	4.00	0.77	0.23	0.22	<loq	0.44	1.23	0.00	0.00	7.73
	MOx	297	220127	-66.92	-69.10	nd	1.51	6.79	1.27	0.68	0.42	<loq	0.71	2.67	0.02	0.00	14.1
	DEEP	621	220127	-66.92	-69.10	0.15	1.94	3.74	0.72	0.63	0.25	<loq	0.41	1.35	0.01	0.00	9.19
**AN2_ST07**	SURF	5	220130	-70.85	-79.89	0.39	0.33	4.00	0.29	0.20	0.09	<loq	0.39	0.40	0.00	0.00	6.09
	DCM	21	220130	-70.85	-79.89	0.30	0.44	3.48	0.46	0.15	1.84	<loq	0.36	0.31	0.01	0.00	7.35
	1%PAR	75	220130	-70.85	-79.89	0.28	0.46	4.44	0.83	0.18	0.22	<loq	0.66	1.64	0.01	0.00	8.72
	MOx	211	220130	-70.85	-79.89	<loq	0.38	3.89	0.69	0.33	0.16	0.75	0.41	1.25	0.00	0.00	7.87
	DEEP	587	220130	-70.85	-79.89	0.28	0.97	3.95	1.20	0.18	0.34	<loq	0.45	1.06	0.00	0.00	8.43
**AN2_ST10**	SURF	5	220201	-67.70	-75.00	<loq	0.69	3.96	0.79	0.11	0.19	<loq	0.38	0.70	0.01	0.00	6.82
	DCM	49	220201	-67.70	-75.00	0.28	1.11	5.03	2.03	0.25	0.16	<loq	0.40	0.76	0.01	0.00	10.0
	1%PAR	200	220201	-67.70	-75.00	<loq	1.81	5.66	1.21	0.13	0.20	<loq	0.50	0.71	0.01	0.00	10.2
	MOx	1000	220201	-67.70	-75.00	0.16	0.47	4.57	0.55	0.23	0.15	<loq	0.65	0.53	0.00	0.00	7.32
	DEEP	2003	220201	-67.70	-75.00	<loq	1.02	4.86	0.85	0.18	0.21	<loq	0.60	0.80	0.01	0.00	8.53
**AN2_ST11**	SURF	5	220204	-62.57	-58.62	nd	1.16	12.57	3.46	0.36	0.24	<loq	1.20	1.01	0.02	0.00	20.0
	DCM	37	220204	-62.57	-58.62	<loq	0.90	3.49	1.59	0.23	0.17	<loq	0.32	1.51	0.01	0.00	8.23
	1%PAR	201	220204	-62.57	-58.62	<loq	0.77	1.97	0.83	0.10	0.08	<loq	0.20	0.33	0.00	0.00	4.30
	MOx	850	220204	-62.57	-58.62	0.14	1.27	2.73	1.15	0.16	0.17	<loq	0.30	0.83	0.03	0.00	6.78
	DEEP	1618	220204	-62.57	-58.62	nd	1.28	3.39	1.60	0.25	0.22	<loq	0.39	1.91	0.34	0.00	9.38

**Category "minor-OPE" results from the sum of: TEHP, TPrP, TPPP, TBPO, TPPO, Chlorpyrifos, DOPP, TDMPP, TDBPP and TTBPP; nd = non detected; <loq = value below limit of quantification;* ∑TCPP = sum of three TCPP isomers (TCPP1, TCPP2, and TCPP3); ∑TcPs = sum of three cresyl isomers (TpCP, TmCP and TpCP). *Stations 10 and 11 showed strong mixing and weak MOx layer.*

References

(1) Estrada, M. (2012) in *Libro blanco de métodos y técnicas de trabajo oceanográfico en la Expedición Malaspina,* 399-345.

(2) Smith, D. C.; Farooq, A (1992). A simple, economical method for measuring bacterial protein synthesis rates in seawater using 3H-leucine. Environmental Science.

(3) Gasol, J. M.; Moran, X. A. G. (2015). Flow Cytometric Determination of Microbial Abundances and Its Use to Obtain Indices of Community Structure and Relative Activity. McGenity, T. J.; Timmis, K. N.; Nogales Fernández, B. Hydrocarbon and Lipid Microbiology Protocols. Biochemical Methods; Springer Protoc. Handbooks, 159-187.
